# Transcriptome and metabolome analysis reveals anthocyanin biosynthesis pathway associated with ramie (*Boehmeria nivea* (L.) Gaud.) leaf color formation

**DOI:** 10.1186/s12864-021-08007-0

**Published:** 2021-09-22

**Authors:** Xinkang Feng, Gang Gao, Chunming Yu, Aiguo Zhu, Jikang Chen, Kunmei Chen, Xiaofei Wang, Aminu Shehu Abubakar, Ping Chen

**Affiliations:** Institute of Bast Fiber Crops, Chinese Academy of Agricultural Sciences, Changsha, 410205 China

**Keywords:** Ramie variety, Leaf, Color formation, Metabolites, Anthocyanins

## Abstract

**Background:**

The bast fiber crop ramie can be used as high-quality forage resources, especially in tropical or subtropical region where there is lack of high-quality protein feed. Hongxuan No.1 (HX_1) is a unique ramie variety with a light reddish brown leaf color, which is obviously different from elite cultivar, Zhongzhu No.1 (ZZ_1, green leaf). While, the regulatory mechanism of color difference or secondary metaboliates synthesis between these two varieties have not been studied.

**Results:**

In this study, phenotypic, transcriptomic and metabolomic analysis of HX_1 and ZZ_1 were conducted to elucidate the mechanism of leaf color formation. Chromaticity value and pigment content measuring showed that anthocyanin was the main metabolites imparting the different leaf color phenotype between the two varieties. Based on LC/MS, at least 14 anthocyanins were identified in leaves of HX_1 and ZZ_1, and the HX_1 showed the higher relative content of malvidin-, pelargonidin-,and cyanidin-based anthocyanins. Transcriptome and metabolome co-analysis revealed that the up-regulated expression of flavonoids synthesis gene was positively correlated with total anthocyanins accumulation in ramie leaf, and the differentfially expression of “blue gene” (F3’5’H) and the “red gene” (F3’H) in leaves bring out HX_1 metabolic flow more input into the cyanidin branch. Furthermore, the enrichment of glycosylated modification pathway (UGT and AT) and the expression of flavonoid 3-O-glucosyl transferase (UFGT), anthocyanidin reductase (ANR), in leaves were significantly influenced the diversity of anthocyanins between HX_1 and ZZ_1.

**Conclusions:**

Phenotypic, transcriptomic and metabolomic analysis of HX_1 and ZZ_1 indicated that the expression levels of genes related to anthocyanin metabolism contribute to the color formation of ramie variety. Anthocyanins are important plant secandary metabilates with many physiological functions, the results of this study will deepened our understanding of ramie leaf color formation, and provided basis for molecular breeding of functional forage ramie.

**Supplementary Information:**

The online version contains supplementary material available at 10.1186/s12864-021-08007-0.

## Background

Ramie is a perennial herb of Urticaceae widely grown in some Asian countries such as southern China, Laos, Thailand and Indonesia [1]. It is nicknamed “crop with the best fiber quality” in China and has been cultivated for thousands of years. In addition to fiber applications, ramie can be used as high-quality forage resources (rude protein > 20%), especially in southern China where there is lack of high-quality protein feed [2]. With a wide range of medicinal value including antioxidant, antibacterial, anti-inflammatory and anti-diabetic proper-ties [3–6] ramie forage is valuable feed that would enhance immunity of livestock and poultry, reduce the drug residue problems caused by drug feed additives and promote healthy development of animal husbandry.

In the national ramie germplasm resource garden nursery of Chinese academy of agricultural sciences, there are more than 2800 kinds of ramie varieties. The leaf color is one of the important phenotypic that can distinguish these different varieties. For example, emerald green color in the elite cultivar Zhongzhu No.1 (ZZ_1) and red leaves and stems in Hongxuan No.1 (HX_1). Previous studies have shown that anthocyanins are the main factor that determined plant color [7]. Usually, the six kinds of common anthocyanins (cyanidin, pelargonidin, delphinidin, peonidin, petunidin, malvidin) are very important for plants [8]. As a branch of phenylalanine pathway, anthocyanin synthesis pathway has been well studied in many species. It is divided into three stages. In the first stage, dihydroflavonols (DHK) is formed by catalytic action of chalcone synthase (CHS), chalcone isomerase (CHI) and flavanone 3-hydroxyxylase (F3H) enzymes. Flavonol synthase (FLS), flavonoid 3′-hydroxylase (F3’H), flavonoid 3′,5′-hydroxylase (F3’5’H), dihydroflavonol-4-reductase (DFR), anthocyanidins synthase (ANS) and other enzymes catalyze the formation of flavonols and cyanidins in the second stage. The third stage, is driven by leucoanthocyanidin reductase (LAR), anthocyanidin reductase (ANR), UDP-glucose: flavonoid 3-O-glucosyltransferase (UFGT) resulting into procyanidins and anthocyanins of various colors.

Recently, the research on the color of leaves and petals was very hot [9, 10]. Transcriptional and metabolic studies were conducted to understand flavonoid metabolism pathway and screen key genes to provide basis for molecular breeding of blue waterlily [11]. The differences of leaf color and “Yin Rhyme” flavor of different tea varieties were caused by differences of anthocyanins, catechins, caffeine, limonene and so on. Their differentially expressed genes were concentrated in the flavonoid metabolism pathway [12]. In fleshy roots, the difference in pigment accumulation in root bark of purple radish and green radish at different developmental stages were linked to anthocyanin biosynthesis rather than flavonol biosynthesis [13]. At present, the effect of anthocyanins on plant color has been extensively studied and some important structural and regulatory genes from different species have been cloned. However, the synthesis and regulation mechanism of anthocyanins in various crops are quite different [11, 12, 14]. The differences observed in leaf color between the ramie varieties ZZ_1 and HX_1 remained at morphological stages with no prior functional / regulatory genes investigated to unravel the reason behind such differences as well as the underlying metabolism pathway culminating in the production of the green and red leaves which has greatly limits further development and application of the new cultivar of HX_1, so the reason of this study.

In addition to play role in plant adaptation and resistance to adversity stresses [15], anthocyanins were also widely used in food [16], feed [17] and pharmaceutical industries [18]. In this study, the color formation related anthocyanins in green leaf variety (ZZ_1) and red leaf variety (HX_1) of ramie were identified and measured. The differentially expressed genes and enrichments pathways for these key components were co-analyzed by transcriptome and metabolomics methods combined with biochemical analysis, fluorescence quantitative PCR and so on. The results of this study will help to provide a theoretical basis for the functional analysis of key genes controlling the color components of ramie leaves, distinctive new varieties breeding.

## Result

### Phenotypes difference between varieties ‘HX_1’ and ‘ZZ_1’

**Fig. 1:**
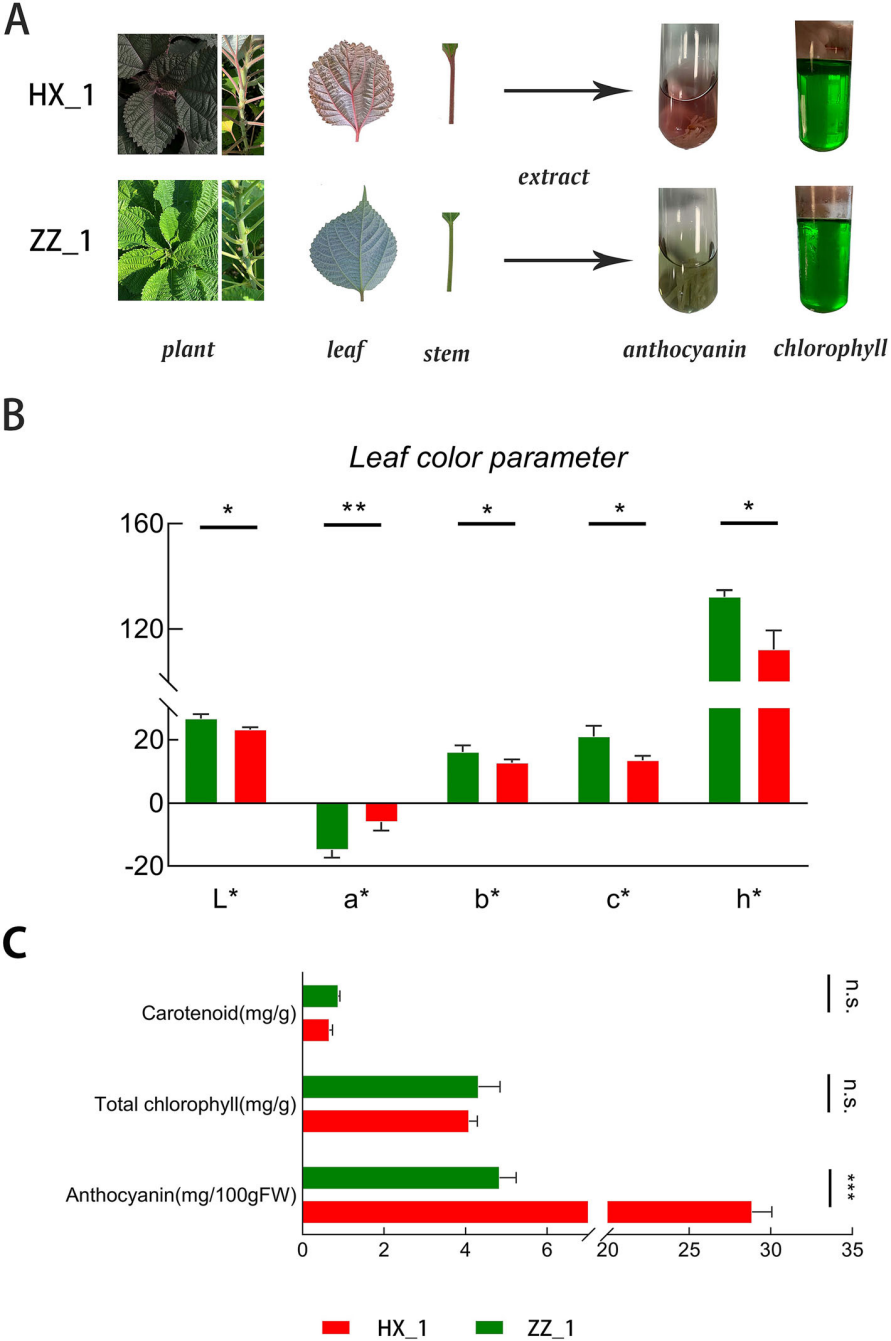
Characteristics of phenotype and pigment content in HX_1 and ZZ_1. (**A**) Phenotypic characteristics of red leaf ramie and green leaf ramie. HX_1 represents the red leaf ramie while ZZ_1 represents the green leaf ramie; (**B**) Variance analysis of leaf color parameters; (**C**) Pigment (Carotenoid, Chlorophyll, Anthocyanin) content and variance analysis; *, **, *** respectively indicates significant difference (*P* < 0.05, *P* < 0.01, P < 0.01), and n. s. indicates no significant difference

The HX_1 and ZZ_1 are two varieties of ramie that can be used as fiber and feed, but their biological characteristics or phenotypes are quite different. Although both of the two varieties have similar leaf outlines, the leaf surface of HX_1 is smoother and the ZZ_1 leaf have more wrinkles. The flower bud, stem and leaf of HX_1 is all red in varying degrees, while the ZZ_1 is bright green (Fig. 1A).

To explore the phenotypic differences between HX_1 and ZZ_1, the leaf color parameters (L^*^, a^*^, b^*^, c^*^, h^*^) of the two varieties were investigated and the indexes of chlorophyll, carotenoid and anthocyanin were determined. The results showed that all the leaf color parameters measured between the two cultivars were significantly different (Fig. 1B). The L^*^ of HX_1 is significantly less than that of ZZ_1, and as L^*^ represents brightness, it implies that ZZ_1 is more colorful. The a^*^ represents red and green concentration of the color, positive value of a^*^ means red, and the negative value green. In this study, the a^*^ values of both samples were negative, and HX_1 showed extremely significantly greater a^*^ value than ZZ_1. According to the a^*^ values, both of the HX_1 and ZZ_1 showed a main color of green, while HX_1 contain more red elements. The b^*^ represents the concentration of yellow and blue elements. Both HX_1 and ZZ_1 had a positive b^*^ value, and the value of ZZ_1 is significantly higher than HX_1. The value of c^*^ (saturation), h^*^ (chromaticity angle) were also positive with the c^*^, h^*^ value of ZZ_1 significantly greater than HX_1.

The quantitative analysis of various pigments showed that chlorophyll is the most abundant pigment, and anthocyanin is the main difference pigment between the two varieties (Fig. 1C). These may be the main reason for the color difference between the two varieties. The anthocyanin content of red variety was significantly (*P* < 0.01) higher (5 times higher) than that of the green leaf ZZ_1. No differences were obtained for chlorophyll and carotenoid content, and chlorophyll was more than 140 times the amount of anthocyanin (mg/g) which signifies that the color tone of ramie leaves as green.

### RNA-Seq and de novo assembly

In order to identify candidate genes that cause leaf color differences, the transcriptome sequencing and the de novo assembly of 8 samples (HX1_1, HX1_2, HX1_3, HX1_4, ZZ1_ 1, ZZ1_ 2, ZZ1_3 and ZZ1_4) from leaves of two varieties under the same conditions were conducted using the ZZ_1 as reference genome [19]. About 54.38 Gb total clean bases were obtained by RNA-seq after cleaning and quality checking, with an average of 6.80 Gb for each sample. The lowest value of Q30 (percentage of bases with sequencing error rate lower than 1 ‰) was 89.96%. The content of GC ranged from 48.76 to 50.02% (Table 1). These data indicated that the sequencing quality is reliable and suitable for further analysis.

**Table 1:** Summary of sequencing data quality preprocessing results

Sample	raw reads	raw bases	clean reads	clean bases	valid bases	Q30	GC
HX1_1	48.04 M	7.21G	44.96 M	6.56G	91.01%	89.68%	49.37%
HX1_2	50.71 M	7.61G	46.96 M	6.81G	89.52%	88.96%	50.02%
HX1_3	49.53 M	7.43G	46.19 M	6.71G	90.25%	89.45%	49.25%
HX1_4	48.01 M	7.20G	44.89 M	6.57G	91.17%	89.28%	49.53%
ZZ1_1	51.12 M	7.67G	47.92 M	7.00G	91.25%	89.85%	48.76%
ZZ1_2	52.71 M	7.91G	49.40 M	7.19G	90.95%	89.93%	48.94%
ZZ1_3	47.44 M	7.12G	44.52 M	6.49G	91.15%	90.04%	49.10%
ZZ1_4	51.21 M	7.68G	48.17 M	7.05G	91.79%	89.93%	49.00%

Subsequently, the filtered clean reads from each sample were mapped to ramie ZZ_1 genome. The mapping rate varied from 91.98% ~ 93.07% (ZZ_1) and 77.28% ~ 86.63% (HX_1), respectively. (Among them, 86.13% ~ 87.17% (ZZ_1) and 71.60% ~ 81.29% (HX_1) were uniquely mapped (Additional file 1: Table S1). The reads number of ‘+’ and ‘-'chains was less than 1% among different ramie varieties and samples (Additional file 1: Table S1). These fragments were used to calculate the mRNA expression level, expressed in FPKM (Fragments Per kb Per Million Reads), to further compare the expression of different genes among different samples. Statistical table of FPKM distribution of mRNA the expression level of all samples tended to be stable (Additional file 2: Table S2). Box plot, density map and regional distribution map of mRNA expression level was shown in Fig. S1A&B&C (Additional file 3). Principal component analysis confirmed the genetic differences in gene expression between the two ramie varieties (Additional file 3: Fig. S1E&F). The sample level clustering of FPKM divided eight samples into two categories (Additional file 3: Fig. S1D).

### GO and KEEG term classification of DEGs in HX_1 vs. ZZ_1

**Fig. 2:**
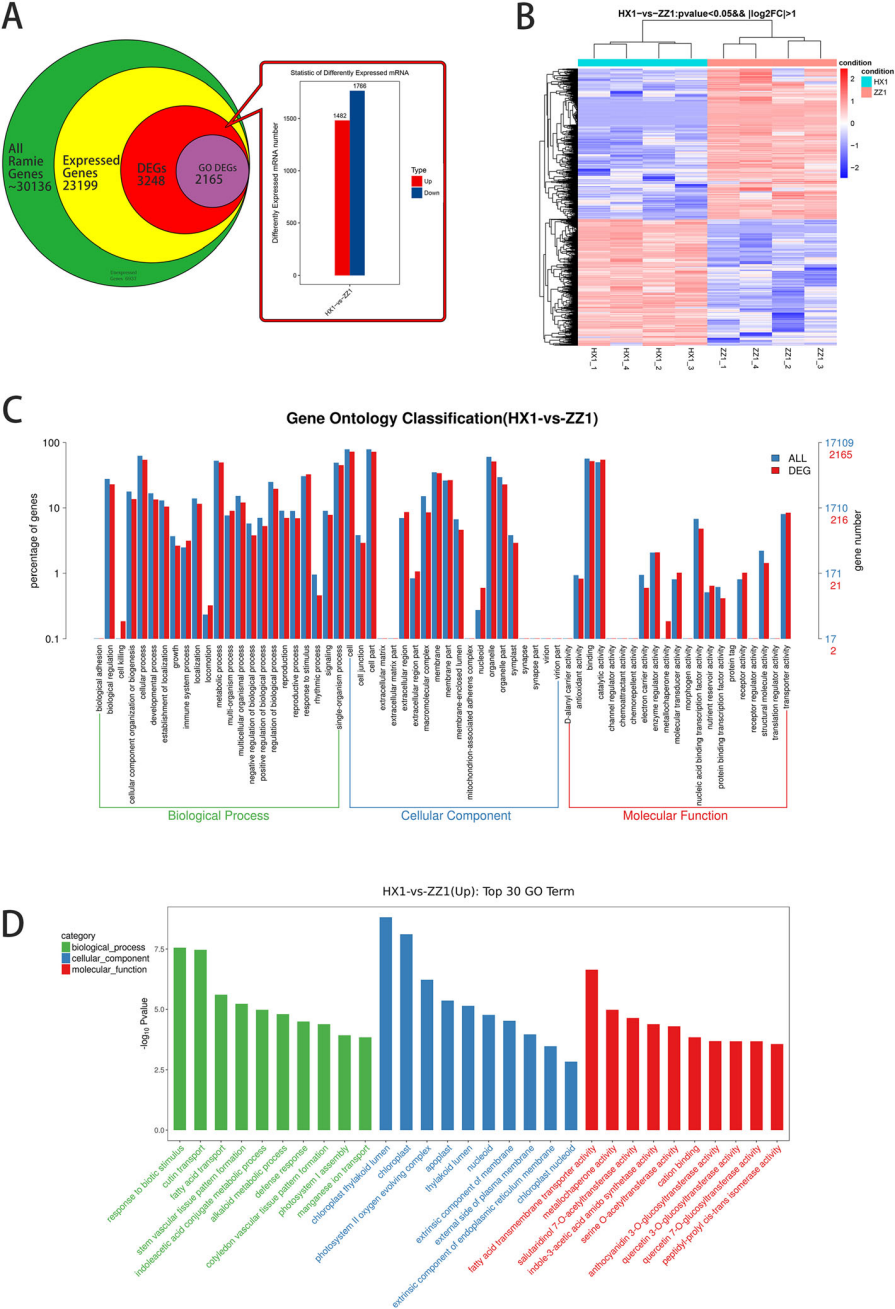
Identification and GO functional enrichment analysis of DEGs. (**A**) Screening of differential genes; (**B**) Cluster analysis of differential genes by thermography; (**C**) Comparison of the distribution of differentially expressed mRNA and all mRNA at GO Level2 level; (**D**) GO enrichment analysis top30 (Up)

**Fig. 3:**
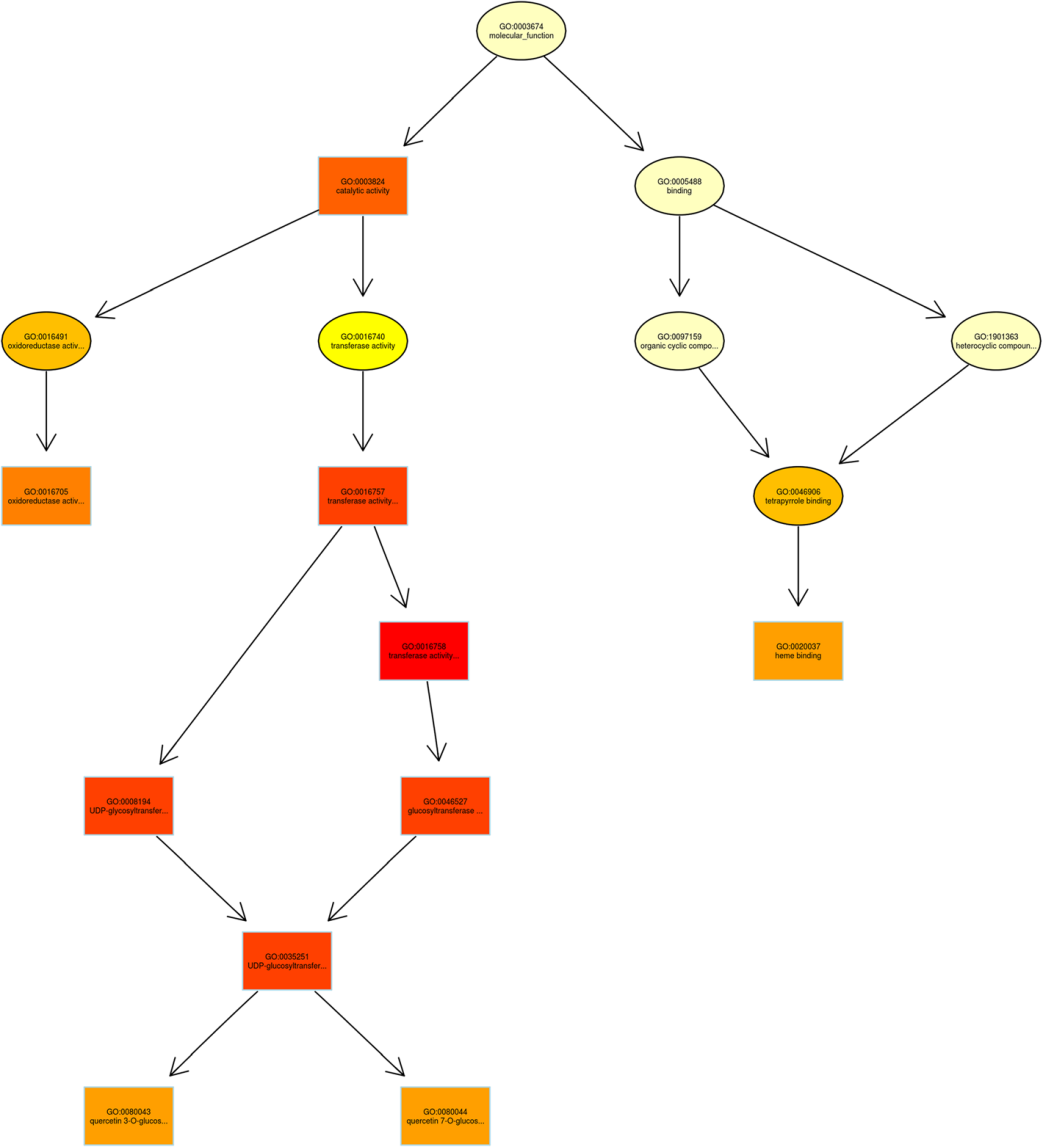
The directed acyclic graph for the enriched terms based on Molecular Function-GO

Based on the false discovery rate (FDR) ≤ 0.05 and fold change (FC) ≥2, a total of 3248 differentially expressed genes (DEGs) were identified (Fig. 2A). Among them, 1482 DEGs were up-regulated and 1766 DEGs were down-regulated in red variety (HX_1) compared with green variety (ZZ_1) (Fig. 2A& B). The overall distribution of DEGs were shown by MA map (Additional file 4: Fig. S2A) and volcano map (Additional file 4: Fig. S2B). Briefly, 2165 DEGs were annotated by 2559 GO terms (Fig. 2C&D) and were divided into 3 groups (BP, CC, MF) (Additional file 5: Table S3). Up regulation of DEGs in the Top10 GO term were enriched in growth and development, organogenesis and biological defense. The expression of flavonoid synthesis related genes was significantly up-regulated (Fig. 2D), which was consistent with the phenotypic data. The directed acyclic graph for the enriched terms based on top-GO showed that UDP glycosyltransferase related branches (GO:0035251, GO0080043, GO0080044) were significantly enriched (Fig. 3).

**Fig. 4:**
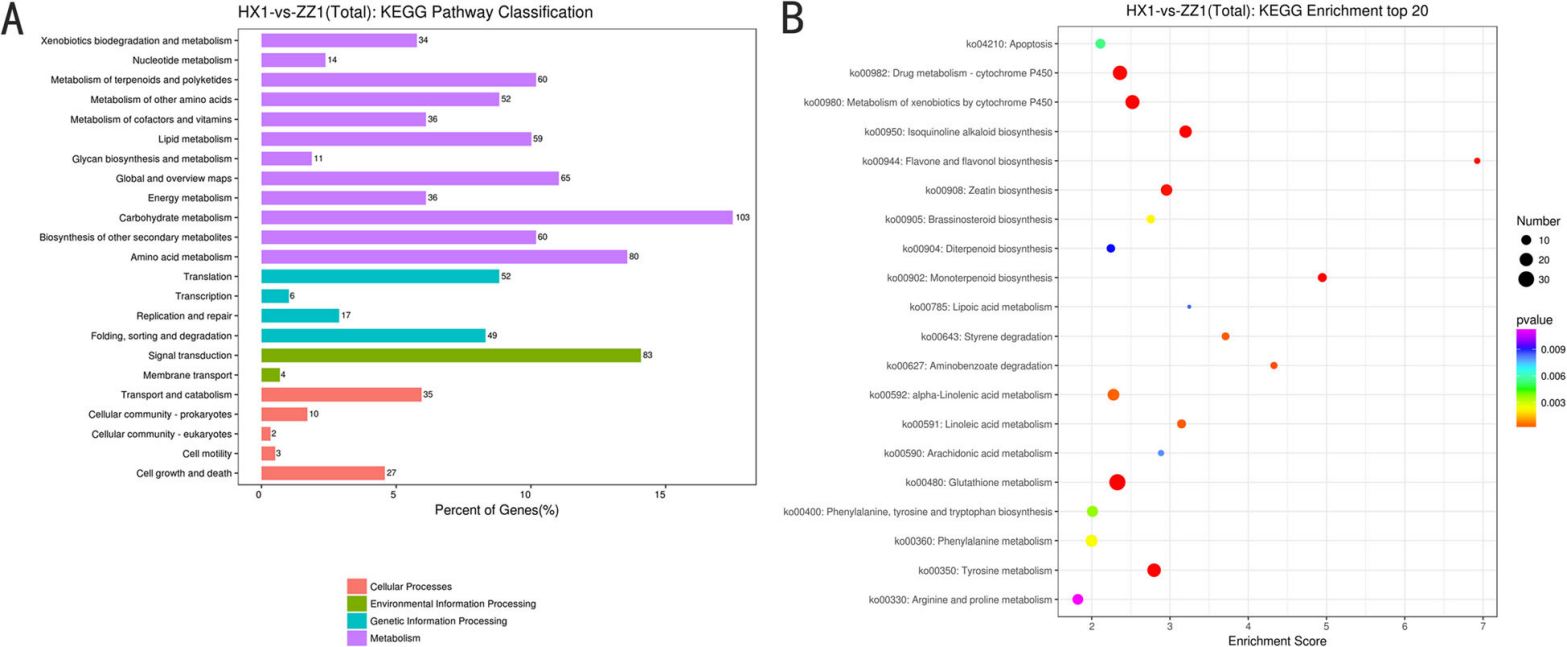
KEGG enrichment analysis of differential mRNA. (**A**) KEGG classification of differentially expressed mRNA; (**B**) KEGG enrichment top20 Bubble Diagram. The larger the bubble size, the more the number of differential mRNA. The color of the bubble changed from purple-blue-green-red. The smaller the value of enrichment *p*-value was, the greater the significance was

KEGG functional enrichment analysis was also carried out to clarify the roles of these DEGs in HX_1 vs. ZZ_1 (Fig. 4A). The number of DEGs in metabolism category was the largest, 181 metabolic pathways were enriched, of which 153 were up-regulated and 157 were down-regulated. Specifically, Phenylalanine, tyrosine and tryptophan biosynthesis (ko00400), Glutathione metabolism (ko00480), Phenylpropanoid biosynthesis (ko00940), Flavonoid biosynthesis (ko00941) and Flavone and flavonol biosynthesis (ko00944) pathways were up-regulated and enriched (Fig. 4B, Additional file 6: Fig. S3). The GO and KEEG analysis of these DEGs provided clues for the comparison of the differences between the two cultivars, especially the molecular events related to leaf color development.

### Anthocyanin metabolism pathway and color formation related genes analyses between HX_1 and ZZ_1

**Fig. 5:**
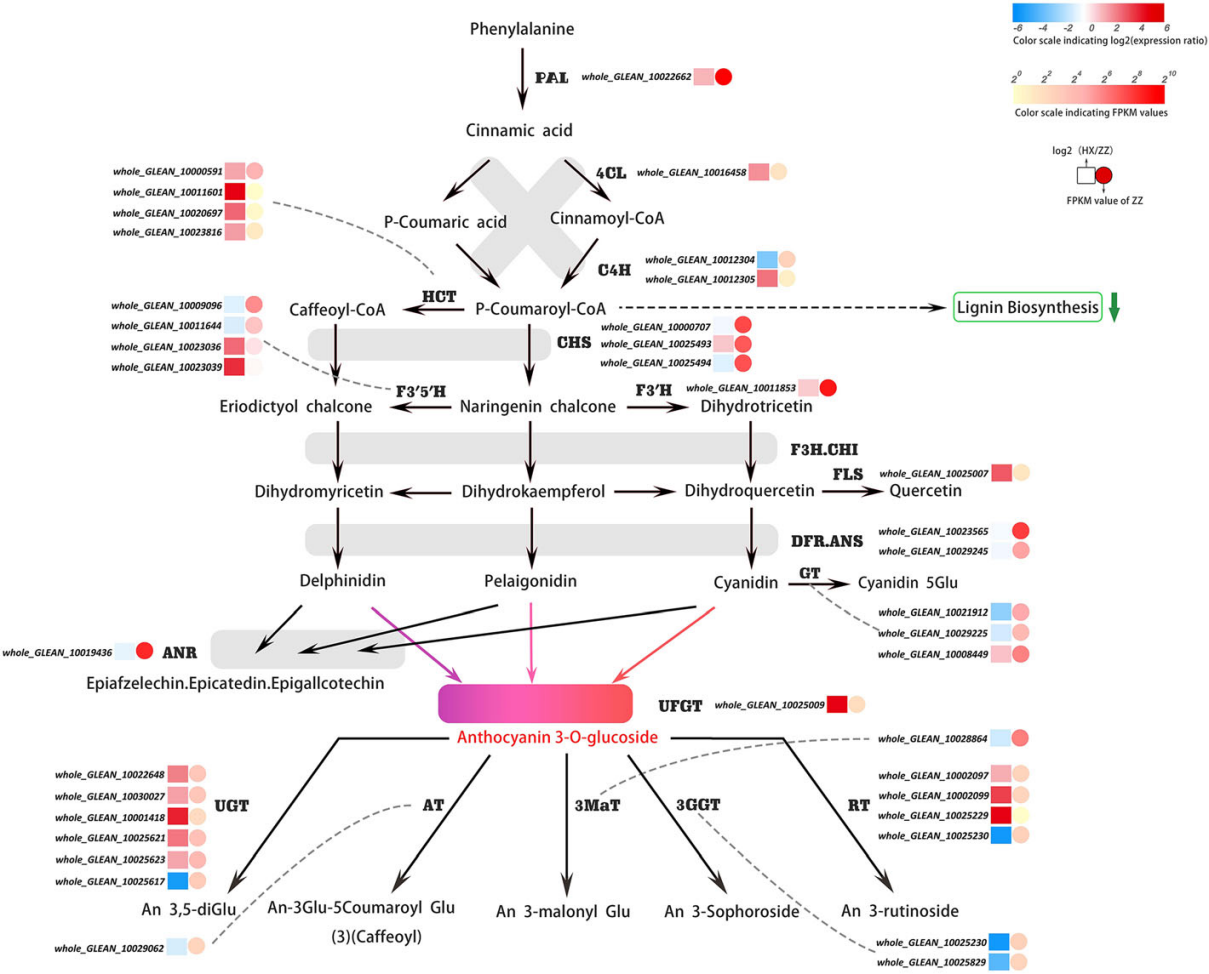
Detailed part of flavonoid metabolic subnetwork showing the subset of nodes or metabolites that constitute the process. The enzyme name, unigene names and expression patterns of each step are placed next to it. The square represents the difference multiple of ZZ_1 and HX_1 expression (log2 (HX_1 / ZZ_1)), the circle represents the FPKM value of ZZ_1, and the depth of color indicates the level of expression and difference. PAL: phenylalanine ammonia-lyase; 4CL: 4-coumarate CoA ligase; C4H: trans-cinnamate 4-monooxygenase; HCT: shikimate O-hydroxycinnamoyltransferase; CHS: chalcone synthase; CHI: chalcone isomerase; F3H: flavanone 3-hydroxylase; F3′H: flavonoid 3′-hydroxylase; F3′5′H: flavonoid 3′5′-hydroxylase; FLS: flavonol synthase; DFR: dihydroflavonol 4-reductase; ANS: anthocyanidin synthase; GT: anthocyanidin 5,3-O-glucosyltransferase; ANR: anthocyanidin reductase; UFGT: anthocyanidin 3-O-glucosyltransferase; UGT: anthocyanidin 3-O-glucoside 5-O-glucosyltransferase; AT: anthocyanidin acyltransferase; RT: rhamnosyl transferase; 3GGT: anthocyanidin 3-O-glucoside 2″-O-glucosyltransferase; 3MaT: anthocyanin 3-O-glucoside-6″-O-malonyltransferase

**Fig. 6:**
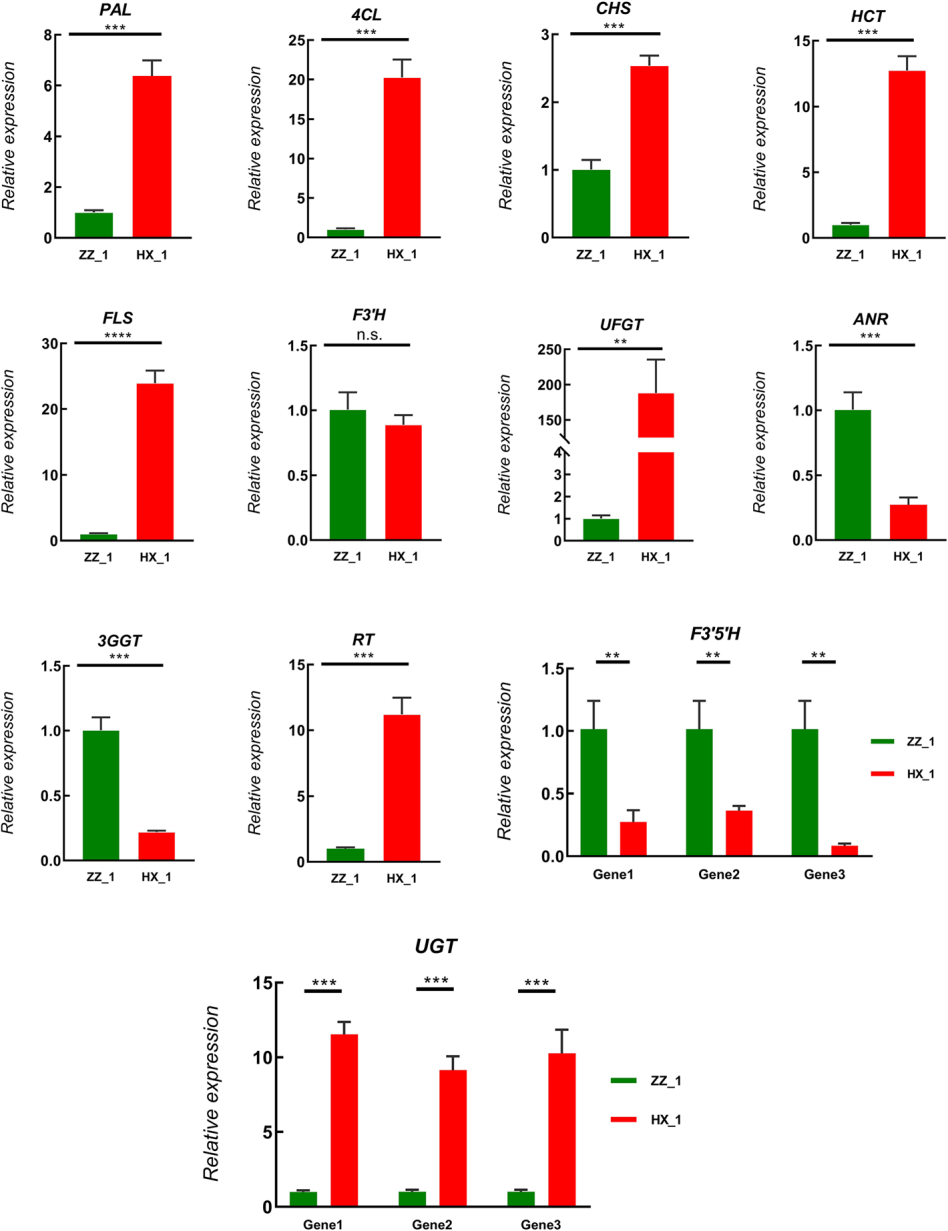
Expression patterns of differentially expressed genes (DEGs) in anthocyanin biosynthesis pathway as determined by q-PCR. Red represents HX_1 and green represent ZZ_1. Data are means ± standard deviations (SD). *, **, *** respectively indicates significant difference (*P* < 0.05, *P* < 0.01, *P* < 0.01), and n.s. indicates no significant difference gene expression value. The outer circle represents the fold change (log_2_FC) of gene expression in HX_1 vs. ZZ_1

Since anthocyanin (ko00942) and its precursor chemical (ko00940; ko00941) synthesis pathways were significantly enriched, their involved DEGs in HX_1 and ZZ_1 was identified and characterized. Most of the related DEGs play role in anthocyanin biosynthesis, and more than half of them had a higher expression level in HX_1 than ZZ_1. These genes could be divided into two groups, one group is the early genes (EGs) including PAL, 4CL, C4H, HCT, CHS, F3H, and the expression level of these genes in HX_1 was higher. The other group is the late gene (LGs), which included the synthetic genes (SGs) and modifying genes (MGs) which play roles in the formation of diverse anthocyanin by group modification such as malonyl, caffeoyl, coumaroyl, succinyl, galloyl, or rhamnosyl at different positions of anthocyanin 3-glucoside. Among these late genes, the UFGT and ANR were significantly up-regulated and the expression level of UFGT gene in HX_1 was almost 26 times higher than in ZZ_1. Another two anthocyanins related genes F3’H and F3’5’H were respectively up- and down-regulated in HX_1 vs. ZZ_1, this may bring out metabolic flow input into the different colored anthocyanins synthesis branches. The modifying genes such as, AT, 3GGT and 3MaT have low basic and negative differential expression levels (Fig. 5). However, most of the UGT (glucosyl or sambubioside were added to the 5 ‘end of the glucoside) and RT (rutinoside was added to the 5 ‘or 3’ end of the glucoside) in the modified genes were significantly up-regulated in HX_1 and ZZ_1 contrast. Fluorescence quantitative PCR (q-PCR) was performed on the mentioned 16 genes to verify the reliability of transcriptome data. As shown in Fig. 6, the expression trends of these genes detected in q-PCR analysis were consistent with those detected in RNA-seq data.

### Metabolomic data analysis (LC/MS)

**Fig. 7:**
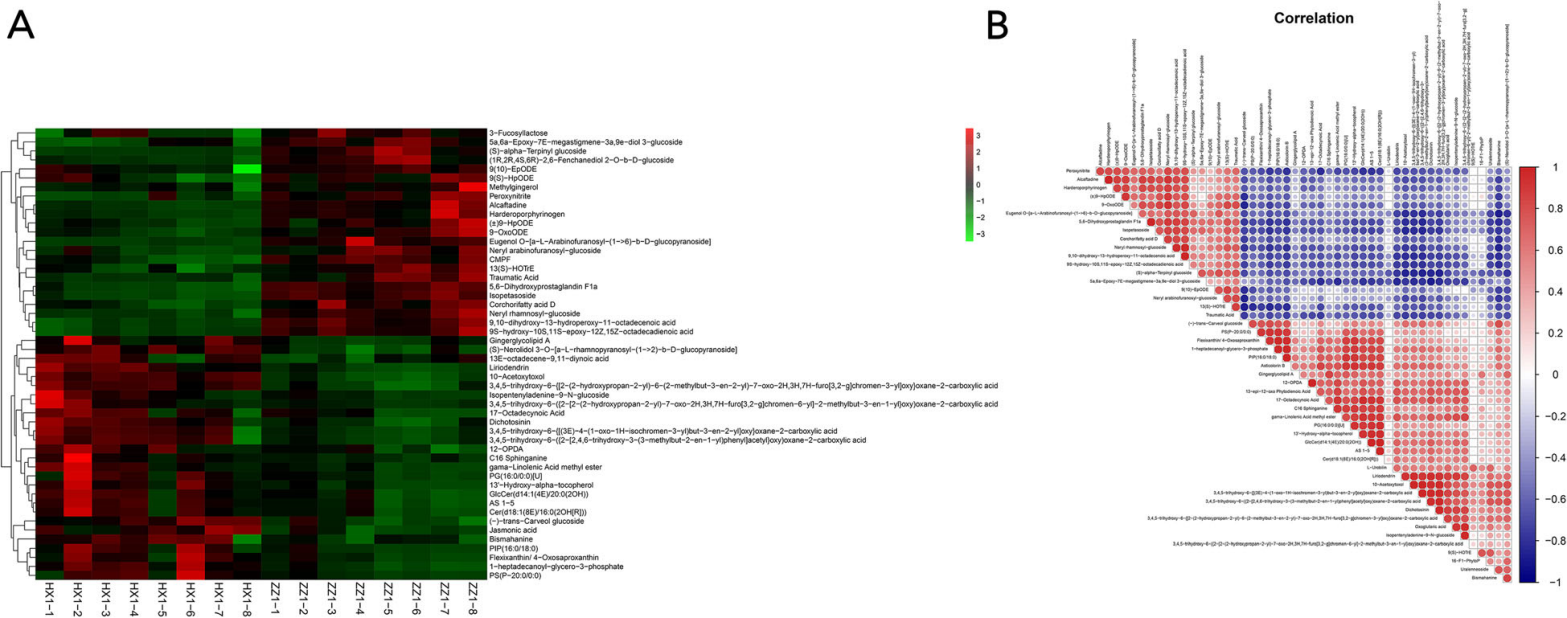
LC-MS: Visual clustering analysis (**A**) and correlation (**B**) analysis of Top50 DEMs

The LC/MS non-targeted metabolomics were conducted for studying of correlation between the differentially expressed genes and the content of metabolites. Firstly, data-sets obtained from UPLC-Triple-TOF-MS were subjected to PCA, PLS-DA (Additional file 7: Fig. S4A; Fig. S4B) to compare the metabolites involved in the color formation of two ramie varieties. The results showed that the two varieties were obviously separated on the PCA score plot corresponding to HX_1and ZZ_1, respectively. Furthermore, OPLS-DA analysis and 200 times response sequencing test of OPLS-DA model were used for modeling the differences between two varieties (Additional file 7: Fig. S4C; Fig. S4D). The metabolites of the differences were selected by statistical analysis (volcano plot, Additional file 7: Fig. S4E) through statistical analysis as described in materials and methods. The general pattern of metabolites is relatively similar in HX_1 and ZZ_1. A total of 5815 metabolites were detected in 20,897 peaks. Among them, 649 metabolites such as flavonoids, anthocyanins and glycerophospholipid were significantly different. According to the VIP value, visual analysis of hierarchical clustering and correlation were performed on the top 50 differential metabolites (Fig. 7), this intuitively shows the interrelation between samples and metabolites among different samples. Flavonoids such as anthocyanin are the most important colorants in plants. As shown in Table 2, there were 15 kinds of modified anthocyanins in the two varieties, which could be classified into four categories. All the identified pelargonidin-, malvidin- and most cyanidin-based anthocyanins showed a high relative content in HX_1 than ZZ_1, whereas delphinidin-based anthocyanins in contrast have a high relative content in ZZ_1 compared to HX_1. We found a total of 9 anthocyanin modifying groups among which the content of MaT-related malonyl modified anthocyanins was found to be much higher in ZZ_1 than in HX_1 (Table 2). The content of AT-related acyl group modified anthocyanins including caffeoyl, coumaroyl, succinyl, oxalyl and galloyl, however, was much higher in HX_1 compared to ZZ_1.

**Table 2:** The anthocyanins in HX_1 and ZZ_1

	m/z	Metabolites	Compound ID	Sub Class	HX_1	ZZ_1	FC
Cyanidin	530.11	Cyanidin 3-(6”succinyl-glucoside)	HMDB0029237	Flavonoid glycosides	43.66	23.64	1.85
	690.14	Cyanidin 3,5-diglucoside (6″,6″‘-malyl diester)	LMPK12010207	Flavonoids	20.58	14.11	1.46
	604.14	Cyanidin 3-sambubioside	HMDB0037976	Flavonoid glycosides	40.97	26.51	1.55
	592.07	Cyanidin 3-(6″-dioxalylglucoside)	HMDB0039936	Flavonoid glycosides	10.07	256.62	0.04
	602.09	Cyanidin 3-(3″,6″-dimalonylglucoside)	HMDB0030096	Flavonoid glycosides	2.38	22.90	0.10
Pelargonidin	674.15	3,5-di-O-(beta-Glucopyranosyl) pelargonidin 6″-O-4, 6″‘-O-1-cyclic malate	LMPK12010067	Flavonoids	16.24	0.79	20.57
Malvidin	332.09	Malvidin	LMPK12010004	Flavonoids	82.16	24.30	3.38
	673.2	Malvidin 3-(6″-p-caffeyglucoside)	LMPK12010384	Flavonoids	28.46	9.85	2.89
	800.22	Malvidin 3-(6-coumaroylglucoside) 5-glucoside	HMDB0038011	Flavonoid glycosides	39.74	7.23	5.50
	784.22	Malvidin 3-p-coumarylrutinoside	LMPK12010385	Flavonoids	40.83	26.73	1.53
Delphinidin	682.14	Delphinidin 3-(6″-malonylsambubioside)	LMPK12010305	Flavonoids	13.27	97.45	0.14
	460.1	4′-O-Methyldelphinidin 3-O-beta-D-glucoside	HMDB0041684	Flavonoid glycosides	88.57	229.22	0.39
	436.1	Delphinidin 3-arabinoside	HMDB0037998	Flavonoid glycosides	4.04	12.44	0.33
	598.15	Delphinidin 3-sambubioside	HMDB0038003	Flavonoid glycosides	15.19	68.13	0.22

Anthocyanin pathway is a branch of flavonoid metabolic pathway; thus, we had also detected many other substances involved in the flavonoid metabolism pathway. As shown in Table 3, a total of 14 flavonoids had been identified, and the content of all flavonoids were found to be higher in HX_1 than ZZ_1, in consistent with the transcriptome data. The flavonol synthase encoded by FLS was the key enzyme leading to flavonoids. The expression level of this gene in HX_1 is 27 times higher than that of ZZ_1.

**Table 3:** The main flavonoids in HX_1 and ZZ_1

m/z	Metabolites	Compound ID	Sub Class	HX_1	ZZ_1	FC
373.1	3′,4′-Methylenedioxy-2,4,6, beta-tetramethoxychalcone	LMPK12020254	Flavonoids	630.56	182.18	3.46
389.1	3-(3,4-dimethoxyphenyl)-5-hydroxy-7-methoxy-8 methyl-3,4-dihydro-2H-1-benzopyran-4-one	HMDB0129573	O-methylated isoflavonoids	1064.87	330.41	3.22
341.1	Brosimacutin D	LMPK12140055	Flavonoids	332.41	41.46	8.02
665.2	Nirurin	LMPK12140614	Flavonoids	2797.60	213.74	13.09
523.2	Dichotosinin	LMPK12020273	Flavonoids	9362.60	4261.89	2.20
663.2	Flavaprenin 7,4′-diglucoside	LMPK12140228	Flavonoids	2945.23	203.81	14.45
538.2	(7′S,8′S)-4,7′-Epoxy-3,8′-bilign-7-ene-3′,5 dimethoxy-4′,9,9′-triol 4′-glucoside	HMDB0040636	2-arylbenzofuran flavonoids	610.02	121.62	5.02
583.2	8-trans-[2-(6-Benzoyloxy-4-hydroxy-2-methoxy-3-methylphenyl) ethenyl]-5-methoxyflavan-7-ol	LMPK12020254	Flavonoids	993.88	278.95	3.56
903.3	Chamuvaritin	LMPK12120473	Flavonoids	4859.48	2062.92	2.36
811.3	3-(3-hydroxyphenyl)-2-phenyl-4-[(E)-2-phenylethenyl] 2,3-dihydro-1-benzofuran-6-ol	HMDB0129161	2-arylbenzofuran flavonoids	188.22	11.14	16.90
551.2	4,6,2′,4′-Tetramethoxychalcone 2′-beta-glucoside	LMPK12120280	Flavonoids	582.56	161.13	3.62
451.1	Afzelechin 7-apioside	HMDB0030824	Flavans	258.82	100.19	2.58
449.1	Koaburanin	LMPK12020253	Flavonoids	540.35	119.89	4.51
739.4	Unanisoflavan	LMPK12080009	Flavonoids	462.82	123.97	3.73

## Discussion

Ramie is traditionally used only as bast fiber resource and more than 80% of its biomass (leaves and young stems) is wasted [2] even though such wasted tender leaf and stem are rich in functional compounds such as flavonoids, chlorogenic acid and many useful bioactive constituents [20]. To harness maximal benefits and ensure multi-purpose utilization of ramie we previously bred two forage ramie variety HX_1 and ZZ_1. Both of these varieties have shown strong regenerative ability and can as well be harvested 4–7 times per year. The main difference between HX_1 and ZZ_1 was their leaf color. Previous studies have shown anthocyanins as the main factor determining plant color, and also an important plant secondary metabolites with many physiological functions [21, 22]. This prompted us to proposed that the variation in the color might not be unconnected to the anthocyanins contents and might affect the quality of HX_1 and ZZ_1 leaves as forage.

Plant pigments determine the color of plants, and the differences in leaf color among different cultivars are due to the relative contents of three main pigment groups (chlorophylls, carotenoids, and anthocyanin) [23]. Analyses of biological characteristics of the ramie varieties ZZ_1 and HX_1 have confirmed that the leaf color of ZZ_1 is green, while that of HX_1 is red. However, precise phenotypic characteristics and the main pigment components and their contents in these varieties had not been determined. The comparison of leaf color parameters showed that there were obvious color differences between the two with the a^*^ value of ZZ_1 being significantly lower than HX_1 which indicated that ZZ_1 is greener. We found that the contents of chlorophyll and carotenoid were similar in the two varieties whereas the anthocyanin content of HX_1 was significantly higher than that of ZZ_1. Anthocyanins are the reason why plant leaves appear red, blue and purple [24, 25]. Though HX_1 has higher anthocyanin content its leaves are green. This is because the chlorophyll content is more than 140 times the anthocyanin content (mg/g). Therefore, we inferred that anthocyanin at low concentrations can’t mask the green color of chlorophylls and color of plants is due to the different relative content of the pigments. These explanations were consistent with those of a previous study on tea [12]. Together, these results indicated that high contents of anthocyanin play critical roles in the redness of leaf of HX_1.

Phenotypic analysis and metabolomics analysis showed that the content of anthocyanins in HX_ 1 leaf was higher than that in ZZ_1. Therefore, the identification of DEGs related to flavonoid and anthocyanin metabolism is a necessary step to elucidating the mechanism of mottled pigment formation. Structural genes involved in flavonoid and anthocyanin biosynthesis have been found and reported in many plants [26]. In our study, PAL, C4H, CHS and other upstream genes were up-regulated, which provided enough precursors for downstream metabolism. Another key node is the bifurcation of anthocyanin synthesis, anthocyanin 3-O glucose transferase (UFGT) encoded by whole_GLEAN_10025009 is the key enzyme in anthocyanin synthesis, also known as BZ1 protein. It is the key step for anthocyanin stability and water solubility in plant [27]. In the grapes, the expression analysis of UFGT genes in white and red-skinned revealed that the UFGT was present, but not expressed in the white skins [28], also exist in apples [29], and tobacco flowers [30]. The loss of function or low expression of UFGT leads to loss or reduced accumulation of anthocyanin [31]. In addition, the expression levels of UGT and RT in anthocyanin modified genes were significantly higher in HX_1 compared to ZZ_1, this did not mean that the transcriptome did not match the metabolome as these modifiers also play a role in the synthesis of other compounds which require further investigation.

The expression level of ramie UFGT gene in HX_1 was almost 26 times than in ZZ_1, these coincides with the anthocyanin accumulation and the leaf color phenotype between HX_1 and ZZ_1. Furthermore, the substrate competitor of UFGT--ANR correlated with epicatechin biosynthesis was strongly up-regulated, which make the synthesis of anthocyanin prior to the biosynthesis of the other substances, leading to high accumulation of anthocyanins in HX_1. Furthermore, F3′H can catalyze the hydroxylation of B-ring at C3′ for various substrates such as dihydrokaempferol and naringenin chalcone, while F3′5′H catalyze the hydroxylation of B-ring at C3′ and C5′, resulting into the production of delphinidin-based anthocyanins. Therefore, F3’5’H and F3’H were known as the “blue gene” and the “red gene” [32]. The two genes were respectively down- and up-regulated in HX_1 vs. ZZ_1, this may bring out HX_ 1 metabolic flow into the cyanidin-based anthocyanin branch, and ZZ_1 into the delphinidin-based anthocyanin branch. Thus, we conclude that the differential expression of UFGT, F3’5’H or F3’H gene were the key factors for anthocyanin accumulation and the leaf color formation in the two ramie varieties.

We also found that anthocyanin synthesis pathway was up-regulated and lignin synthesis pathway significantly down-regulated in HX_1 which was consistent with the metabolome data. Interestingly, the content of lignin decreased and the content of anthocyanins increased, which made the leaves of forage ramie more palatable. Anthocyanin accumulation in ramie may be a determinant for its utilization as forage. We think the results of our study will accelerate the breeding of functional forage ramie variety and their application in tropical, subtropical regions where there is lack of high-quality protein feed.

## Materials and methods

### Plant materials and experimental conditions

The green variety Zhongzhu No.1 and red variety Hongxuan No.1, bred by the Institute of bast fiber crops, Chinese Academy of Agricultural Sciences, were selected as experimental materials in Wangcheng District, Changsha City, Hunan Province. From top to bottom, 4–5 leaves and petioles were collected from ramie plants in the first season under the same environment at the same time. After labeling, they were put into self-sealed bags and put in liquid nitrogen for cryopreservation immediately and later stored at − 80 °C until further experiments.

### LC-MS analysis

The above experimental materials were selected for LC-MS metabolomics analysis. All chemicals and solvents were analytical or HPLC grade. Water, methanol, acetonitrile, formic acid was purchased from CNW Technologies GmbH (Düsseldorf, Germany) and L-2-chlorophenylalanine from Shanghai Hengchuang Bio-technology Co., Ltd. (Shanghai, China). Metabolomics was detected by OE Bio-technology Co., Ltd. (Shanghai, China). Firstly, 80 mg of the sample was weighed, and 20 μl of internal standard (L-2-chlorophenylalanine, 0.3 mg/ml; Lyso PC17:0, 0.01 mg/ml, all methanol configuration) and 1 ml methanol: water (V: V = 7:3) were added. Two small steel balls were then inserted, adding, precooled to − 20 °C for 2 min followed by grinding (60 Hz, 2 min). Following centrifugation, the solution was filtered into LC bottle using 0.22 μ m organic phase pinhole filter and stored at − 80 °C until LC-MS analysis. Quality control samples (QC) were prepared by mixing aliquots of the all samples and used as pooled sample.

The experimental instrument was a liquid chromatography-mass spectrometry (LC-MS) system composed of WatersTM ACQUITY UPLC ultra performance liquid phase tandem AB Triple TOF 5600 high resolution mass spectrometers. The column temperature was 45 °C, mobile phase A-water (containing 0.1% formic acid), B-acetonitrile / methanol (2 / 3) (V / V) (containing 0.1% formic acid), 0.4 ml / min flow rate and 5 μl injection volume. ESI was used as the ion source, and the positive and negative ion scanning mode was used to collect the MS signal. The data preprocessing was basically the same as GC / MS with a difference being only in baseline filtering. Peak identification, integration, retention time correction, peak alignment and normalization were performed by Progenesis QI v2.3 software (Nonlinear Dynamics, Newcastle, UK) for LC-MS raw data. The parameter settings were slightly different, and the metabolites were annotated by the Human Metabolome Database and METLIN metabolite database.

### Identification of differentially expressed metabolites (DEM)

The data matrix was imported into SIMCA software package (14.0, Umetrics, Umeå, Sweden). We used a variety of statistical analysis methods (PCA, PLS-DA, OPLS-DA) to distinguish the overall difference of metabolic profiles between groups and find the metabolites differences between groups. The metabolites with VI*P* value greater than 1.0 and P value less than 0.05 were identified as differentially expressed metabolites (DEMs) by OPLS-DA model and t test of normalized peak area respectively. The *p* value and fold change value obtained by t-test and multiple variation analysis respectively were visualized and volcano map was made to screen differential metabolites. To prevent over fitting, seven cycles cross validation (7-fold cross validation) and 200 response permutation testing (RPT) were used to evaluate the quality of the model.

The KEGG ID of different metabolites was used for pathway enrichment analysis to obtain the enrichment results of metabolic pathway. Compared with the whole background, the hypergeometric test was used to find the pathway items which were significantly enriched in the DEMs. With a *p*-value ≤0.05 set threshold, the smaller it is, the more significant the difference of the metabolic pathway.

### Transcriptome sequencing analysis

After extracting total RNA and digesting DNA with DNase, eukaryotic mRNA was enriched with magnetic beads Oligo (dT). The cDNA was synthesized with six base random primers using the interrupted mRNA as template. The purified double-stranded cDNA was subjected to terminal repair (3′ end plus A base), and then connected with sequencing connector and PCR amplification carried out. After passing the quality inspection with Agilent 2100 Bioanalyzer, the library was sequenced using Illumina HiSeqTM 2500 sequencer.

We used Trimmmatic software to preprocess the quality of the original data (to remove adapter, low-quality reads and low-quality bases). Hisat2 was used to compare Clean Reads with the reference genome of ramie (*Boehmeria nivea* (L.) Gaud.) to obtain the position information on the reference genome or gene, as well as the specific sequence characteristics of the sequencing samples. The reference transcripts were used as the database, and the expression abundance of each transcript in each sample was calculated by sequence similarity comparison using Bowtie and eXpress software.

The expression of mRNA was calculated by FPKM (Fragments Per kb Per Million Reads) method. We used DESeq software to standardize the number of mRNA counts in each sample (using baseMean value to estimate the expression level). Finally, differential mRNA was screened according to the difference multiple (|log2 (foldchange) | ≥1) and difference significance test results (*P* < 0.05).

We performed GO enrichment analysis of differentially expressed mRNA in order to describe its function. The hypergeometric distribution was used to test whether the GO function set was significantly enriched (P < 0.05), and Fisher algorithm was used to analyze the difference mRNA among samples by CC, BP and MF. We used KEGG database to analyze the pathway of differential mRNA (combined with KEGG annotation results), and calculate the significance of differential mRNA enrichment in each pathway entry by hypergeometric distribution test, so as to find out which cell pathway changes may be related to the differential mRNA in different samples [33]. The transcriptome sequencing and analysis were conducted by OE biotech Co., Ltd. (Shanghai, China).

### Combined analysis of transcriptome and metabolome

According to the data of gene expression and metabolite response intensity, Pearson correlation algorithm was used to calculate the correlation between gene expression and metabolite response intensity data. DEGs and DEMs were selected to draw correlation heat map and correlation matrix. According to the results of association analysis between different genes and different metabolites, association network diagram was drawn. At the same time, the differential genes and metabolites pathway were analyzed, and their common pathway information was mapped to KEGG by using the program written by OE.

### Determination of chlorophylls, carotenoids, and anthocyanin contents

Chlorophyll (Chl) and carotenoids (Car) were extracted from ramie leaves with ethanol acetone mixture (1:1). Fresh ramie leaves (0.1 g) were cut into 1 mm strips and put into the test tubes containing 20 ml of the mixture. The tubes were sealed and extracted overnight at room temperature (Shaking 3–4 times during the period). The next day, after centrifugation at 10000 g for 10 min, absorbance was detected at 470 nm, 645 nm and 663 nm by spectrophotometer. Each sample was analyzed in triplicate. The contents of Chl and Car were calculated according to the following formula described by Porra [34] and Parsons and Strickland [35]:

$$ \mathrm{Chl}\ \mathrm{a}\ \left(\mathrm{mg}/\mathrm{g}\right)=\left[\left(12.72{\mathrm{A}}_{663}-2.69{\mathrm{A}}_{645}\right)\ast \mathrm{V}\right]/\left(1000\ast \mathrm{M}\right) $$

$$ \mathrm{Chl}\ \mathrm{b}\ \left(\mathrm{mg}/\mathrm{g}\right)=\left[\left(22.88{\mathrm{A}}_{645}-4.68{\mathrm{A}}_{633}\right)\ast \mathrm{V}\right]/\left(1000\ast \mathrm{M}\right) $$

$$ \mathrm{Total}\ \mathrm{Chl}\ \left(\mathrm{mg}/\mathrm{g}\right)=\left[\left(20.19{\mathrm{A}}_{645}+8.04{\mathrm{A}}_{633}\right)\ast \mathrm{V}\right]/\left(1000\ast \mathrm{M}\right) $$

$$ \mathrm{Car}\left(\mathrm{mg}/\mathrm{g}\right)=\left[\left(8.73{\mathrm{A}}_{470}+2.11{\mathrm{A}}_{663}-9.06{\mathrm{A}}_{645}\right)\ast \mathrm{V}\right]/\left(1000\ast \mathrm{M}\right) $$

Where V is the volume of the extract (ml) and M is the mass of the sample (g).

Anthocyanins were extracted from two samples according to the method described by Guo [12] with slight modification. The content of anthocyanins was determined by extinction coefficient method [36]: 0.1 g fresh leaves was ground into powder in liquid nitrogen and put it into test tube, 10 ml of mixture (1.5 mol/l HCl: 95% Ethanol = 15:85) was added, sealed, extraction performed in dark for 24 h, shaking several times during the period. The mixture was later centrifuged at 4000 r/min for 10 min, supernatant filtered, and volume fixed to 20 ml. Absorbance was measured at 535 nm, and 657 nm with water as a reference. Anthocyanin was then computed using the formula described as follows [36]:

Total anthocyanin content (mg/100gFW) = 100A_max_V/98.2 M

$$ {\mathrm{A}}_{\mathrm{max}}={\mathrm{A}}_{530}-0.25\ast {\mathrm{A}}_{657} $$

Where V is the volume of the extract (ml) and M is the mass of the sample (g).

### Relative expression analyses of selected key DEGs

Total RNA was extracted using EasySpin Plus plant RNA rapid extraction Kit (Aidlab Biotechnologies Co., Ltd) from different plants. To determine the relative transcript levels of the differentially expressed genes the RNA was used as templates and reverse-transcribed using RevertAid First cDNA Synthesis Kit (Thermo Scientific) into cDNA. qRT-PCR analyses were conducted using gene-specific primers and the enzyme and fluorescent dye required for qRT-PCR were quantified by mixed 2 × Sybr Green qPCR Mix (Aidlab Biotechnologies Co., Ltd).

The two-step qRT-PCR had a 25 μl reaction system consisting of 12.5 μl 2 × SYBR qPCR Mix, 1 μl gene-specific primers (forward primer 0.5 μl and reverse primer 0.5 μl), 1.0 μl DNA template, and ddH2O up to 25 μl. CFX96 Touch Deep Well Real-Time PCR System (Bio-Rad) was used to detect gene transcripts. The qRT-PCR reaction procedure included initial denaturation at 95 °C for 3 min, and a total of 39 cycles (95 °C for 10 s, 60 °C for 30 s) was used for denaturation and annealing. Finally, the melt curve program was used to detect the specificity of primers. 18 s gene was used as internal control. Relative transcript levels were calculated using the 2-ΔΔCt formula. The primer sequences were designed using Primer 6 software (Additional file 8: Table S4). All qRT-PCR analyses were performed with three biological and technical replications. All histograms were drawn using GraphPad Prism 8 software and merged using Adobe Photoshop (2020) software.

### Statistical analyses

Each experiment was set up with three biological repeats, and all data were expressed as mean ± standard deviation (SD). Difference between varieties using one-way ANOVA (T Test) and Duncan’s test. The difference between groups is indicated by *. The number of * represents the degree of difference, n.s. represents no significant difference. The data were analyzed using SAS9.4 software.

## Conclusions

In this study, phenotypic, transcriptomic and metabolomic analysis of Hongxuan No.1 and Zhongzhu No.1 revealed various pigments, metabolites (flavonoids, anthocyanin), and metabolic pathways (anthocyanin metabolism) responsible for the leaf color and secondary metabolite profiles of HX_1 and ZZ_1. HX_1 leaf contains higher content of anthocyanin, flavonoids than ZZ_1. High contents of anthocyanin and flavonoids contribute to the deeper red leaf color of HX_1 than ZZ_1. High transcript levels of these genes which involved in anthocyanin metabolism pathways corresponded to increased accumulation of anthocyanin in HX_1. This study will help for the functional analysis of key genes controlling the color components of ramie leaves, and provide a theoretical basis for the breeding of forage ramie and multi-purpose utilization of ramie varieties.

## Supplementary Information

**Additional file 1 Table S1: **Statistical results of comparison rate between reads and reference genome.

**Additional file 2 Table S2**: Statistical table of FPKM distribution of mRNA.

**Additional file 3 Fig. S1:** Analysis of mRNA expression level. **A** is distribution of mRNA expression in 8 samples; **B** is Box plot.

**Additional file 4 Fig. S2:** MA map and Volcano map. (**A**) MA map of differential expression; (**B**) Volcano map of differential expression.

**Additional file 5 Table S3**: GO functional analysis and enrichment classification.

**Additional file 6 Fig. S3:** Top20 enrichment analysis of KEGG. (**A**) HX1-vs-ZZ1 (Up); (**B**) HX1-vs-ZZ1 (Down).

**Additional file 7 Fig. S4:** LC-MS data analysis. (**A**) PCA; (**B**) PLS-DA; (**C**) OPLS-DA analysis; (**D**) 200 times response sequencing test of OPLS-DA model; (**E**) volcano plot.

**Additional file 8 Table S4**: Primer sequences of qRT-PCR analyses.

## Data Availability

The raw data has been uploaded to the National Center for Biotechnology Information Short Read Archive (https://www.ncbi.nlm.nih.gov/sra/PRJNA717370). The accession numbers: SRR14074422; SRR14074423.

## Abbreviations

DEGs: Differentially expressed genes
FPKM: Fragments per kilobase of exon per million fragments
GO: Gene ontology
mRNA: messenger RNA
RNA-Seq: RNA sequencing
qRT-PCR: Quantitative real-time polymerase chain reaction
BP: Biological process
CC: Cellular component
MF: Molecular function
