# Supplementary material for: Transcriptome and metabolome analysis reveals anthocyanin biosynthesis pathway associated with ramie (Boehmeria nivea (L.) Gaud.) leaf color formation
Source: BMC Genomics. 2021 Sep 22;22:684. doi: 10.1186/s12864-021-08007-0 (PMC8456610; doi:10.1186/s12864-021-08007-0)
Supplement: Supplementary file 3 — Additional file 3 Fig. S1: Analysis of mRNA expression level. A is distribution of mRNA expression in 8 samples; B is Box plot. [file 12864_2021_8007_MOESM3_ESM.docx]

**Figure S1:** Analysis of mRNA expression level. A is distribution of mRNA expression in 8 samples; B is Box plot.


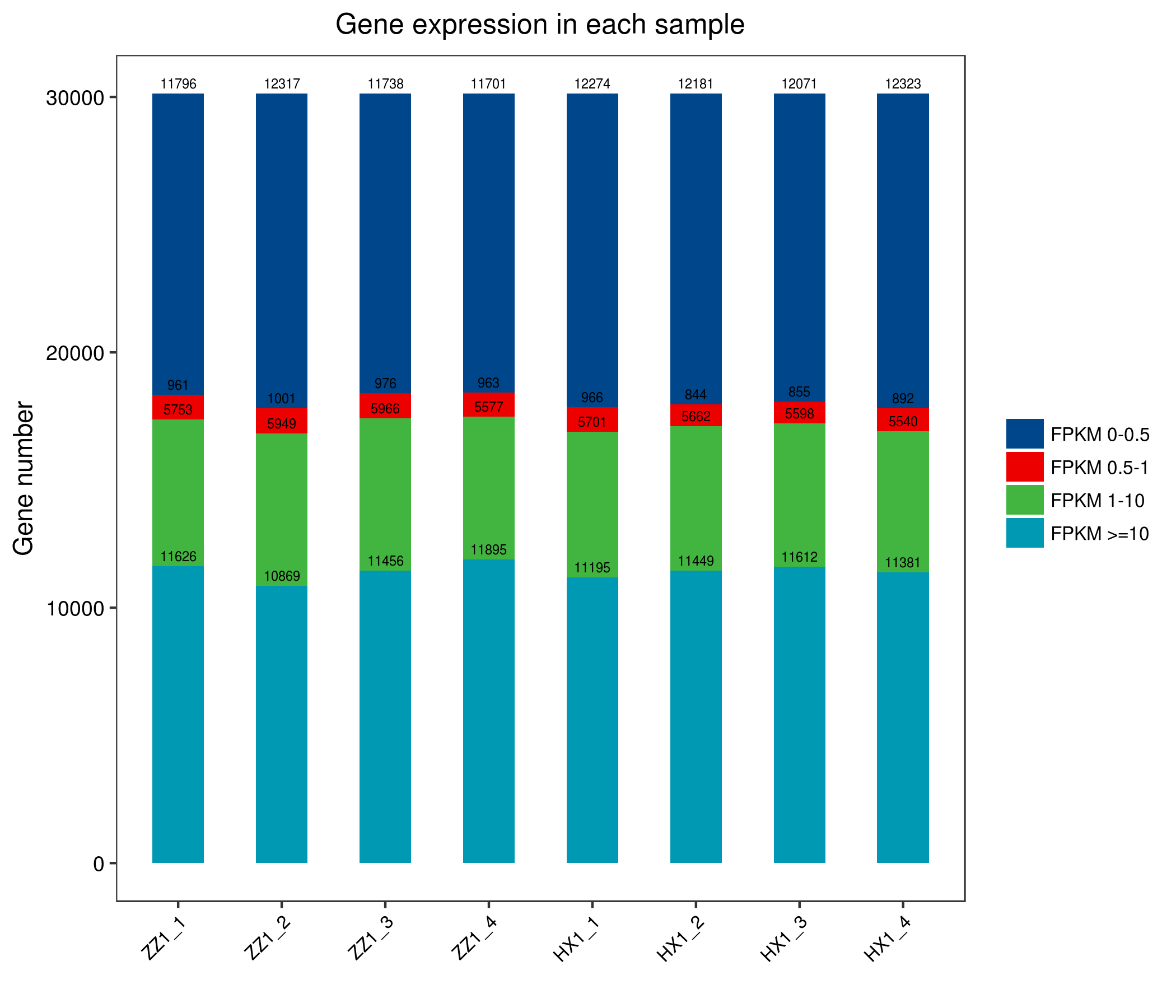


A


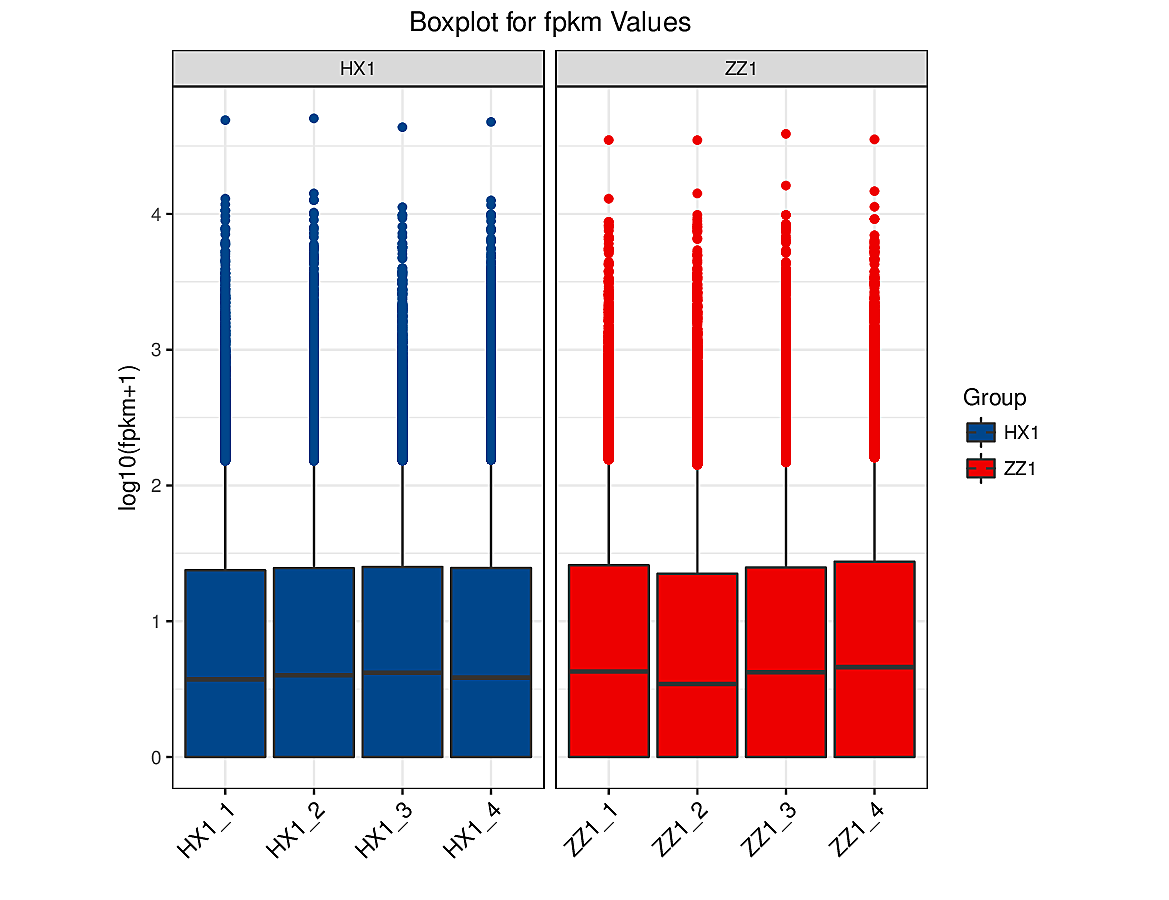


B

**Figure S1:** Analysis of mRNA expression level. C is density map; D is results of sample-to-sample cluster analysis about FPKM.


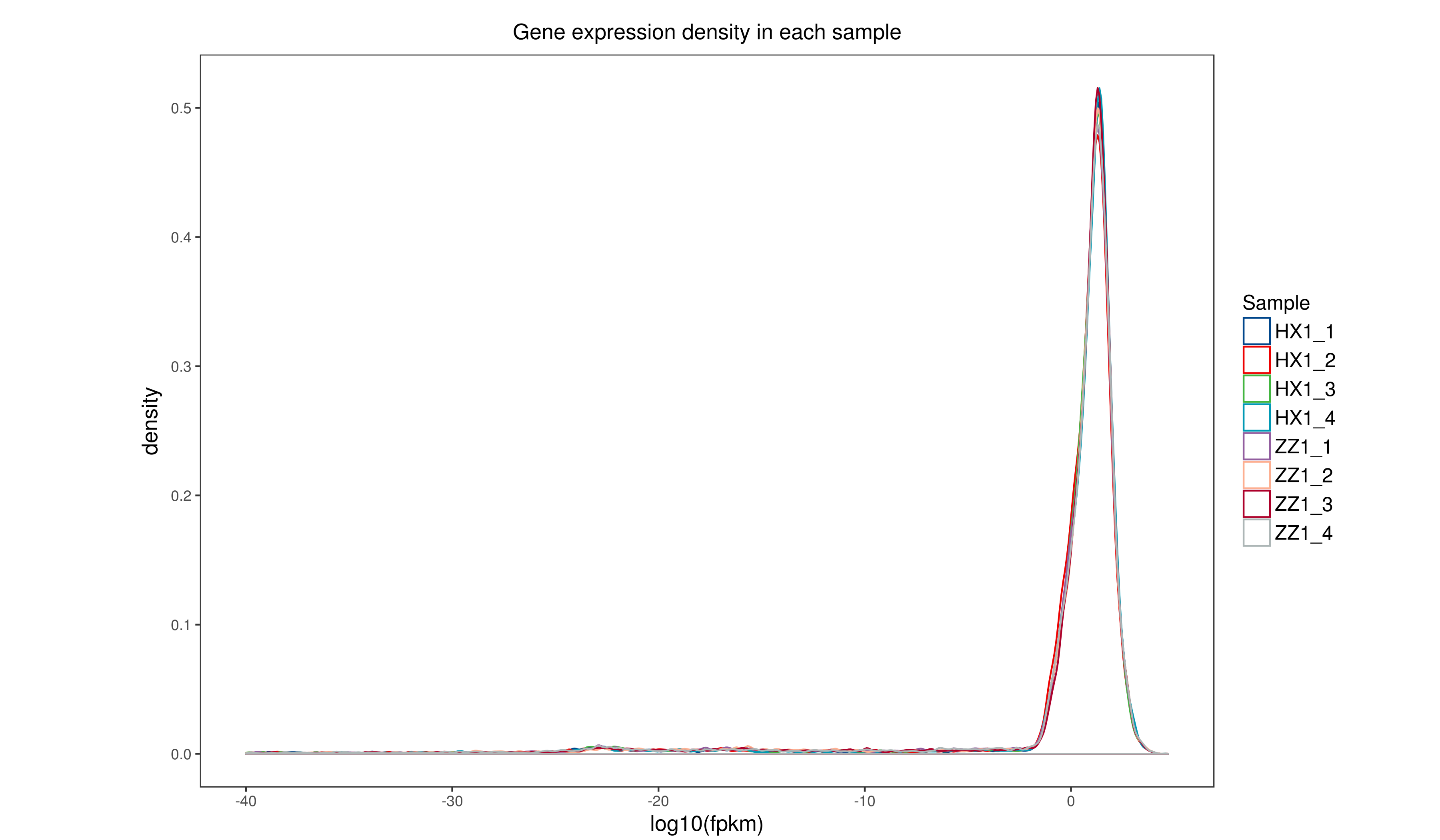


C


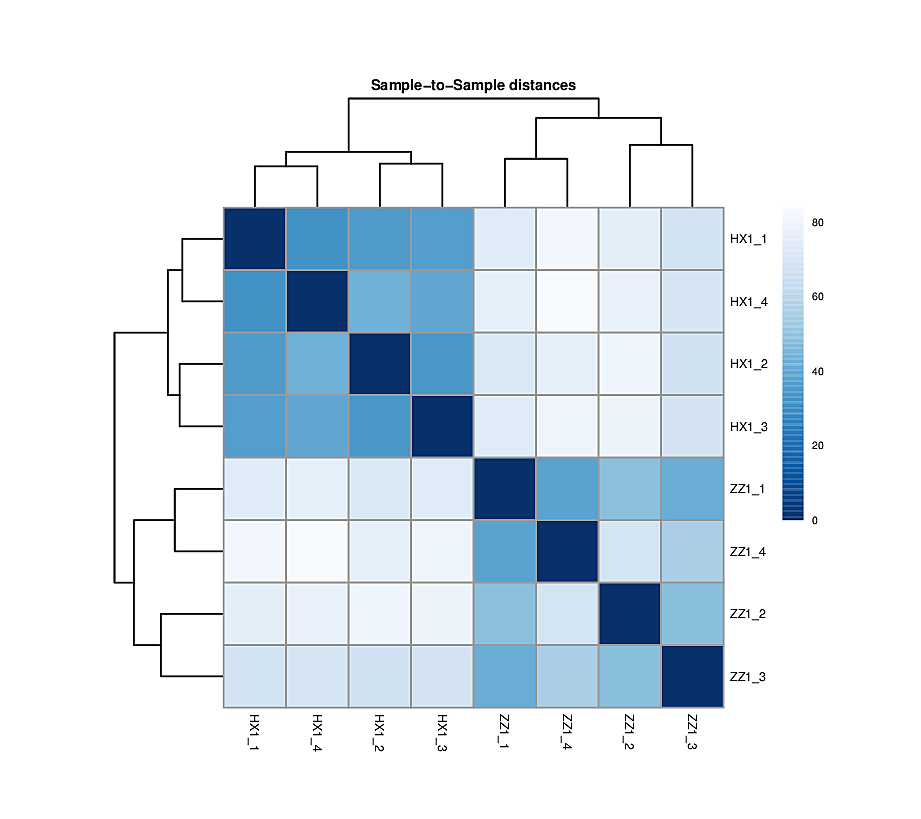


D

**Figure S1:** Analysis of mRNA expression level. E is 3D principal component analysis (PCA); F is 2D PCA.


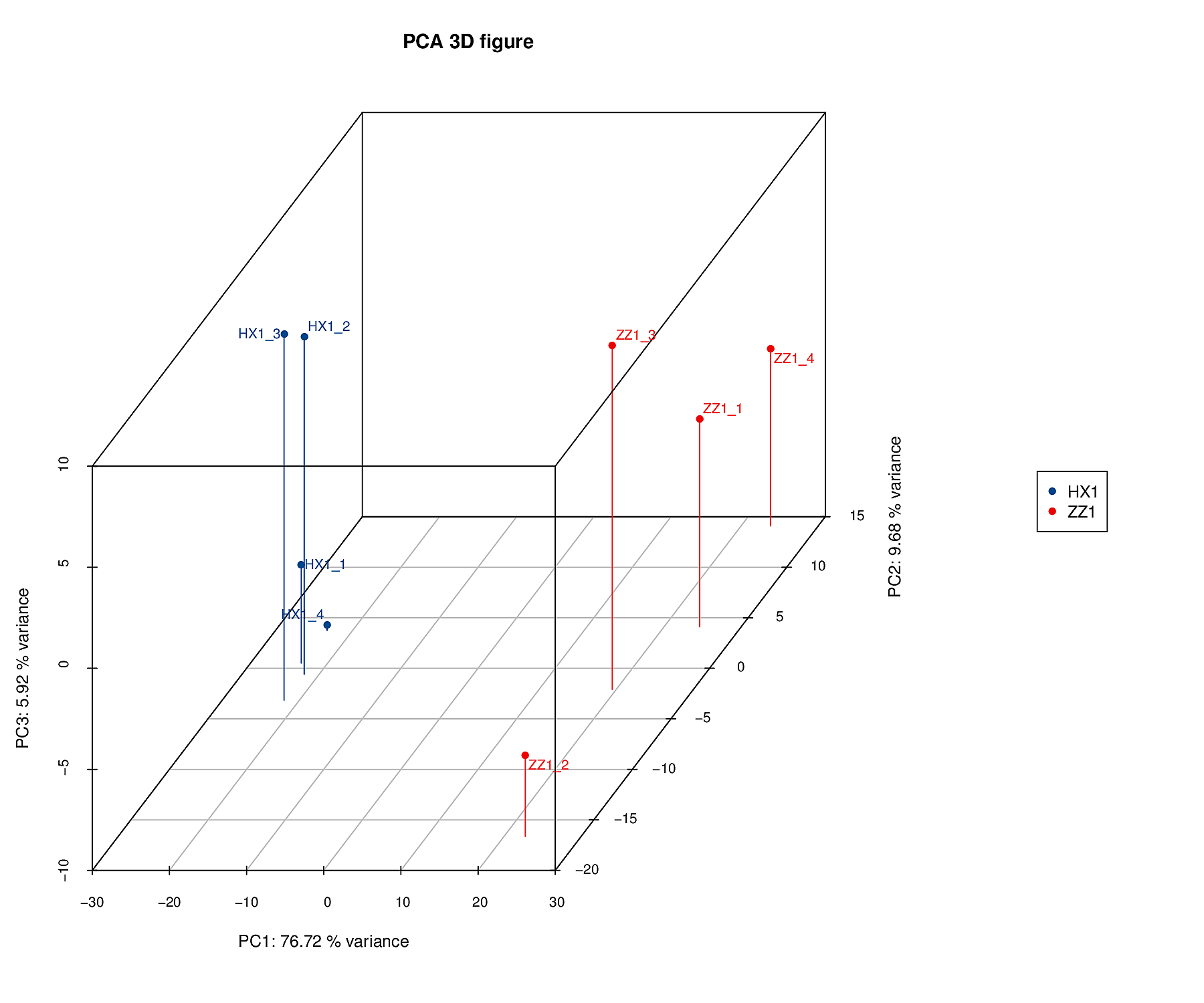


E


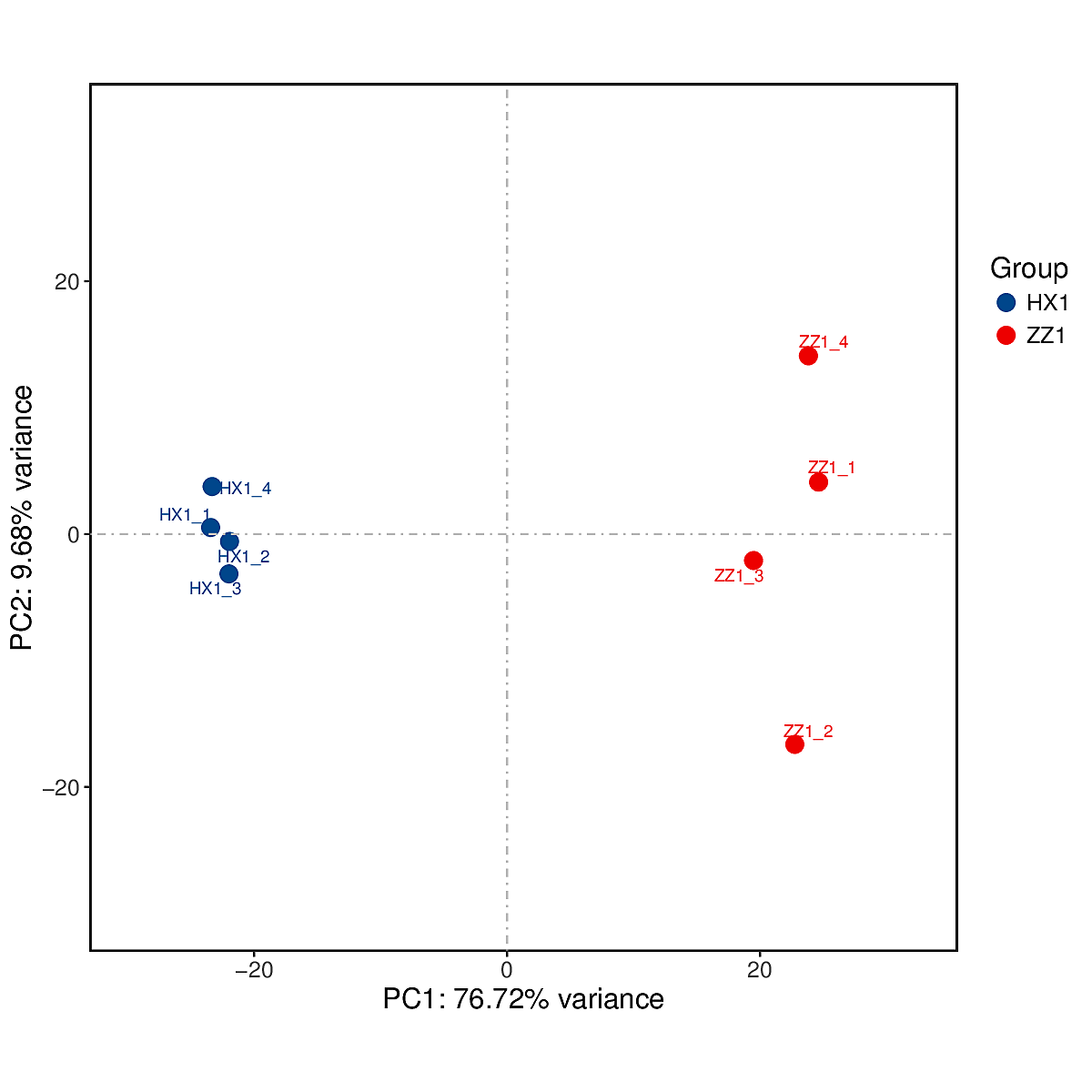


F
