# Supplementary material for: Transcriptome and metabolome analysis reveals anthocyanin biosynthesis pathway associated with ramie (Boehmeria nivea (L.) Gaud.) leaf color formation
Source: BMC Genomics. 2021 Sep 22;22:684. doi: 10.1186/s12864-021-08007-0 (PMC8456610; doi:10.1186/s12864-021-08007-0)
Supplement: Supplementary file 4 — Additional file 4 Fig. S2: MA map and Volcano map. (A) MA map of differential expression; (B) Volcano map of differential expression. [file 12864_2021_8007_MOESM4_ESM.docx]

**Figure S2:** MA map and Volcano map. (A) MA map of differential expression; (B) Volcano map of differential expression.


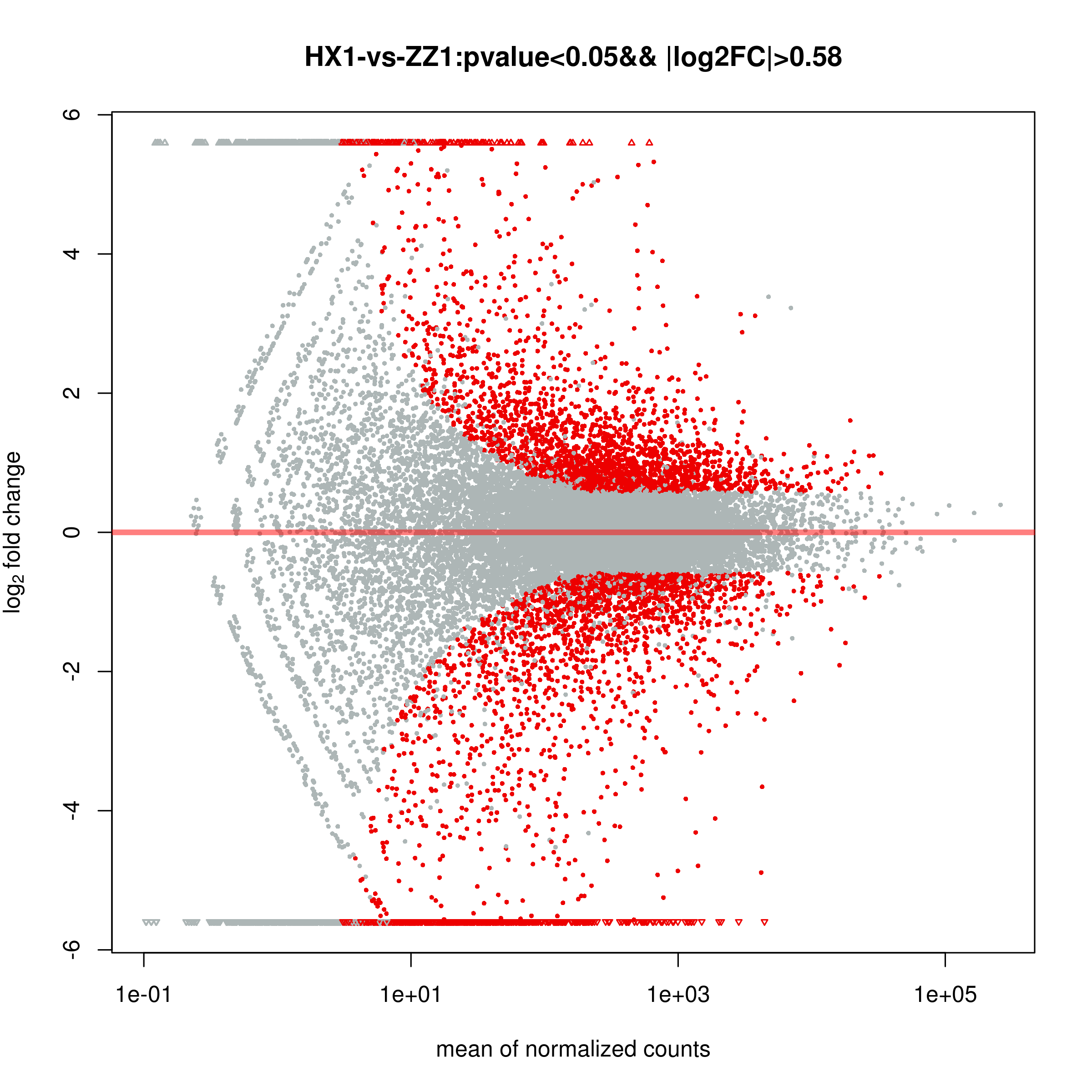


**A**


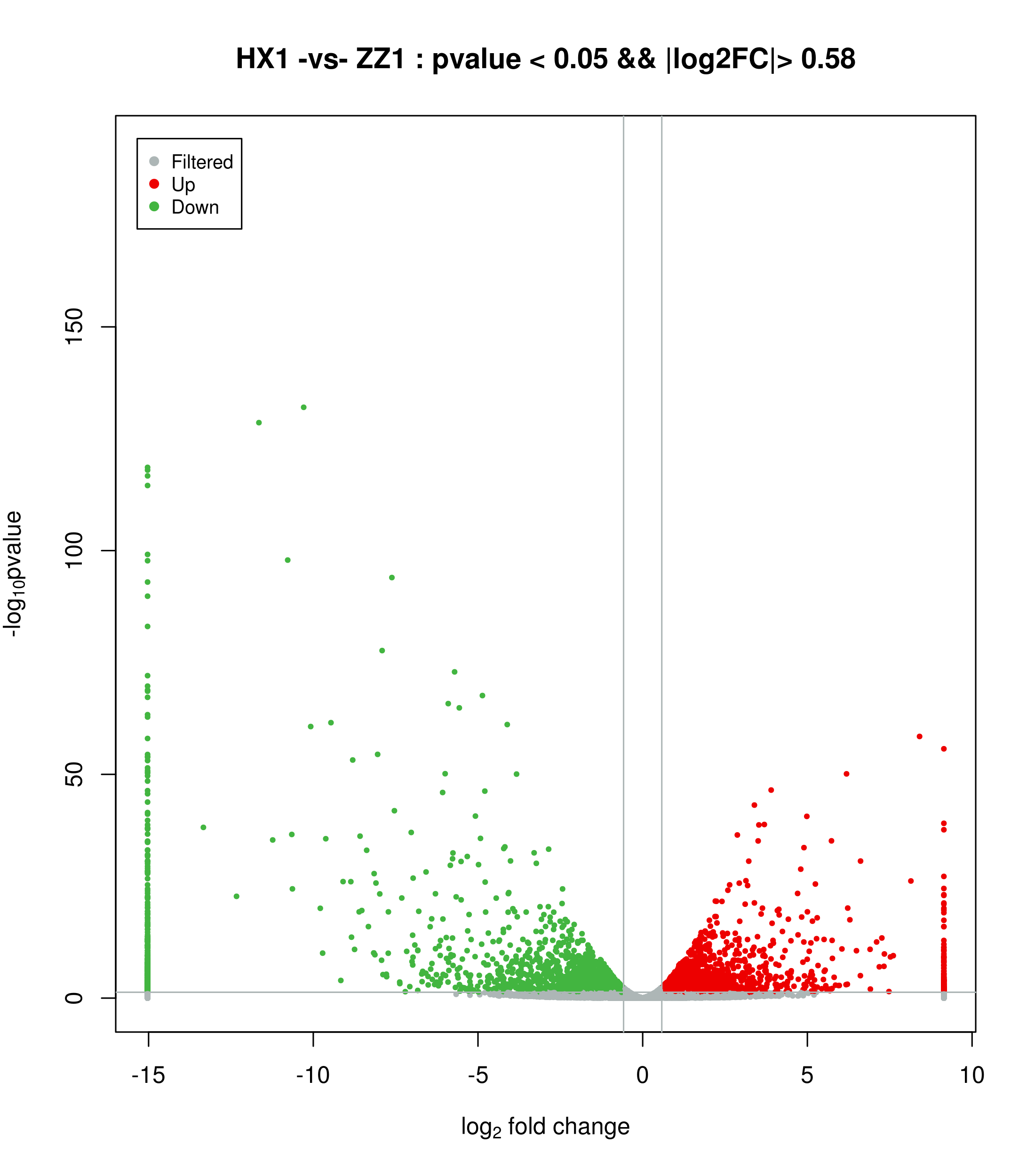


**B**
