# Supplementary figures and images for: Transcriptome and metabolome analysis reveals anthocyanin biosynthesis pathway associated with ramie (Boehmeria nivea (L.) Gaud.) leaf color formation
Source: BMC Genomics. 2021 Sep 22;22:684. doi: 10.1186/s12864-021-08007-0 (PMC8456610; doi:10.1186/s12864-021-08007-0)

**Figure S3:** Top20 enrichment analysis of KEGG. (A) HX1-vs-ZZ1 (Up); (B) HX1-vs-ZZ1 (Down).


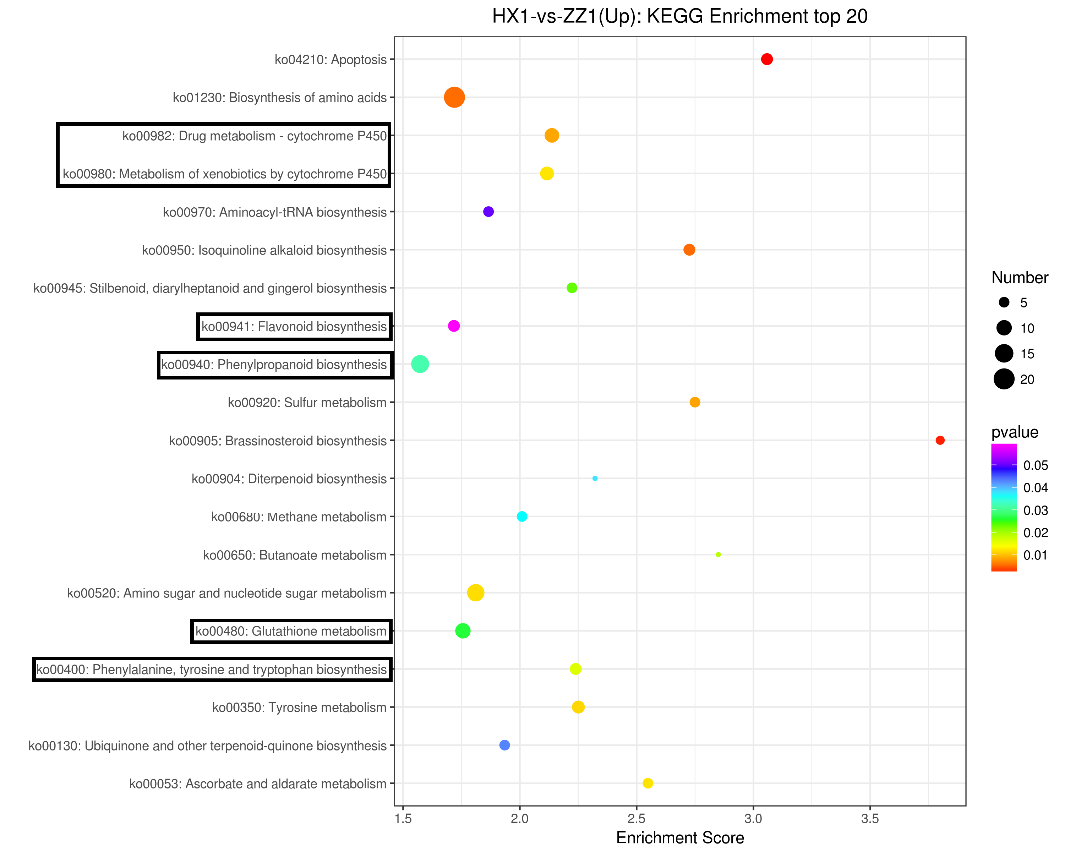


**A**


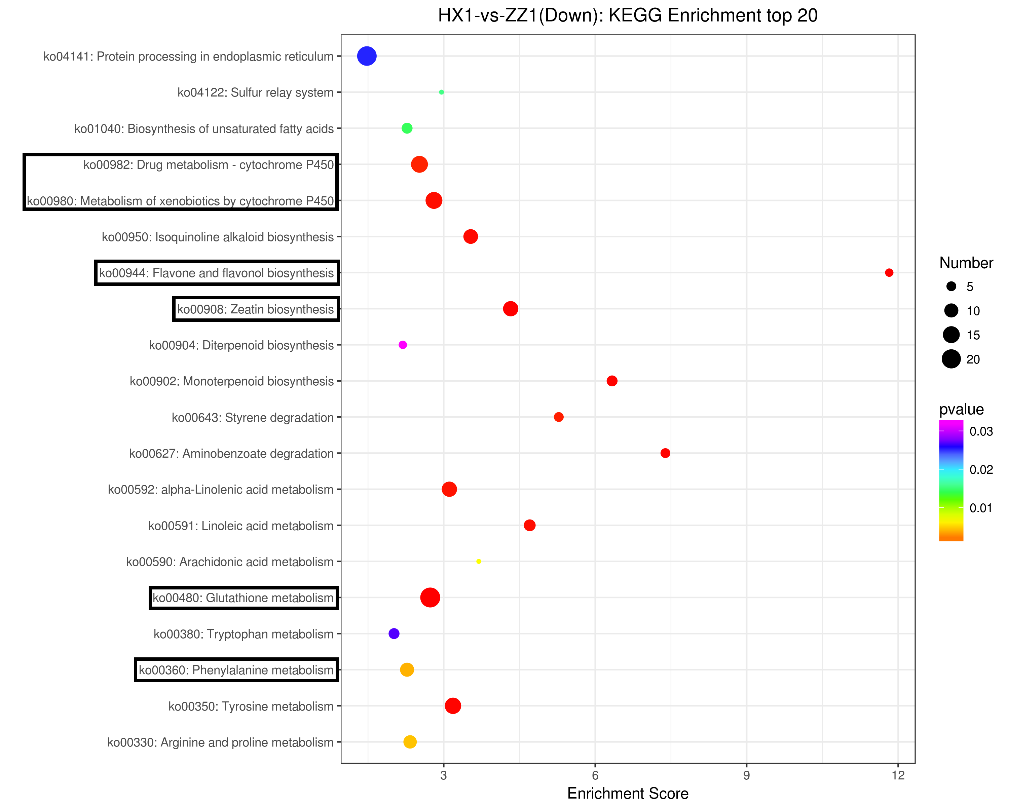


**B**

Supplement: Supplementary file 6 — Additional file 6 Fig. S3: Top20 enrichment analysis of KEGG. (A) HX1-vs-ZZ1 (Up); (B) HX1-vs-ZZ1 (Down). [file 12864_2021_8007_MOESM6_ESM.docx]
