# Supplementary material for: Transcriptome and metabolome analysis reveals anthocyanin biosynthesis pathway associated with ramie (Boehmeria nivea (L.) Gaud.) leaf color formation
Source: BMC Genomics. 2021 Sep 22;22:684. doi: 10.1186/s12864-021-08007-0 (PMC8456610; doi:10.1186/s12864-021-08007-0)
Supplement: Supplementary file 7 — Additional file 7 Fig. S4: LC-MS data analysis. (A) PCA; (B) PLS-DA; (C) OPLS-DA analysis; (D) 200 times response sequencing test of OPLS-DA model; (E) volcano plot. [file 12864_2021_8007_MOESM7_ESM.docx]

**Figure S4:** LC-MS data analysis. (A) PCA; (B) PLS-DA; (C) OPLS-DA analysis; (D) 200 times response sequencing test of OPLS-DA model; (E) volcano plot.

**A**


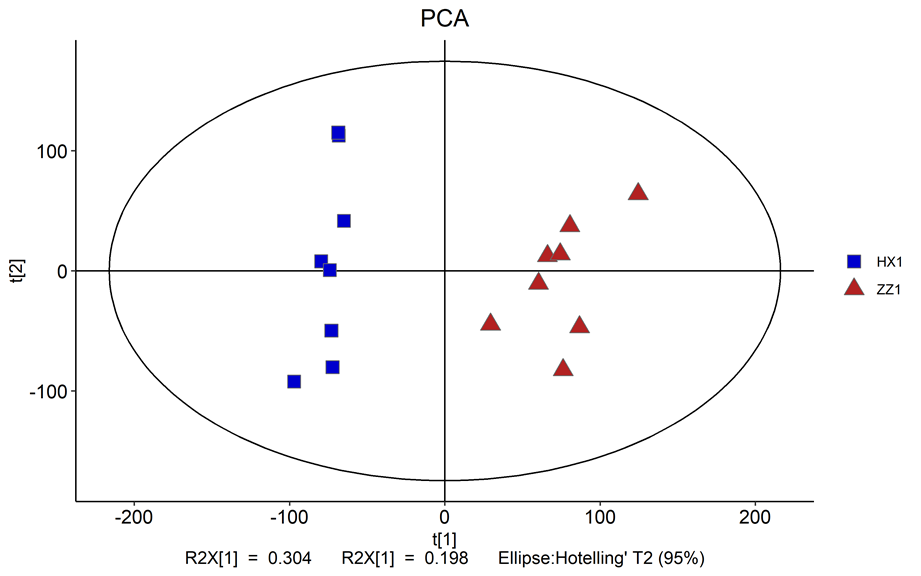


**B**


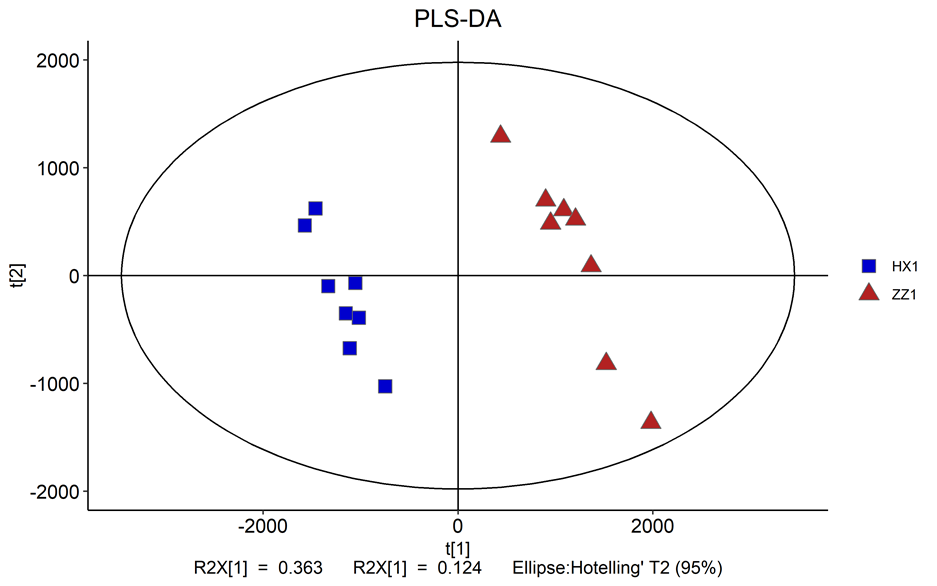


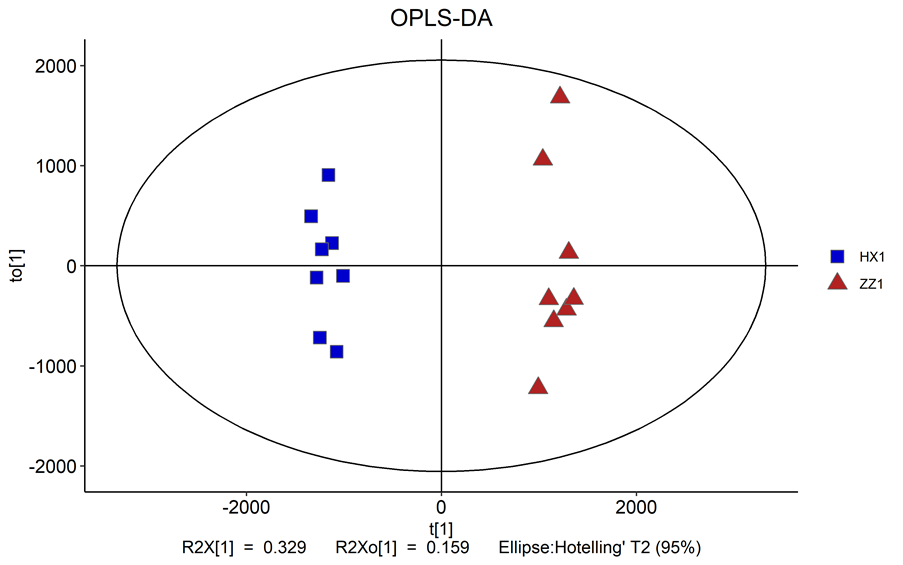


**C**

**D**


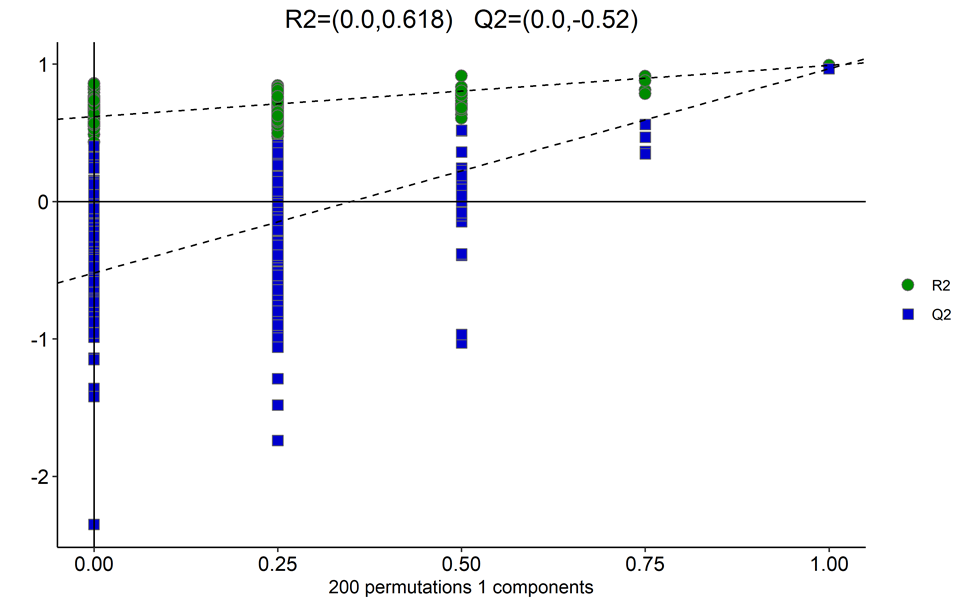


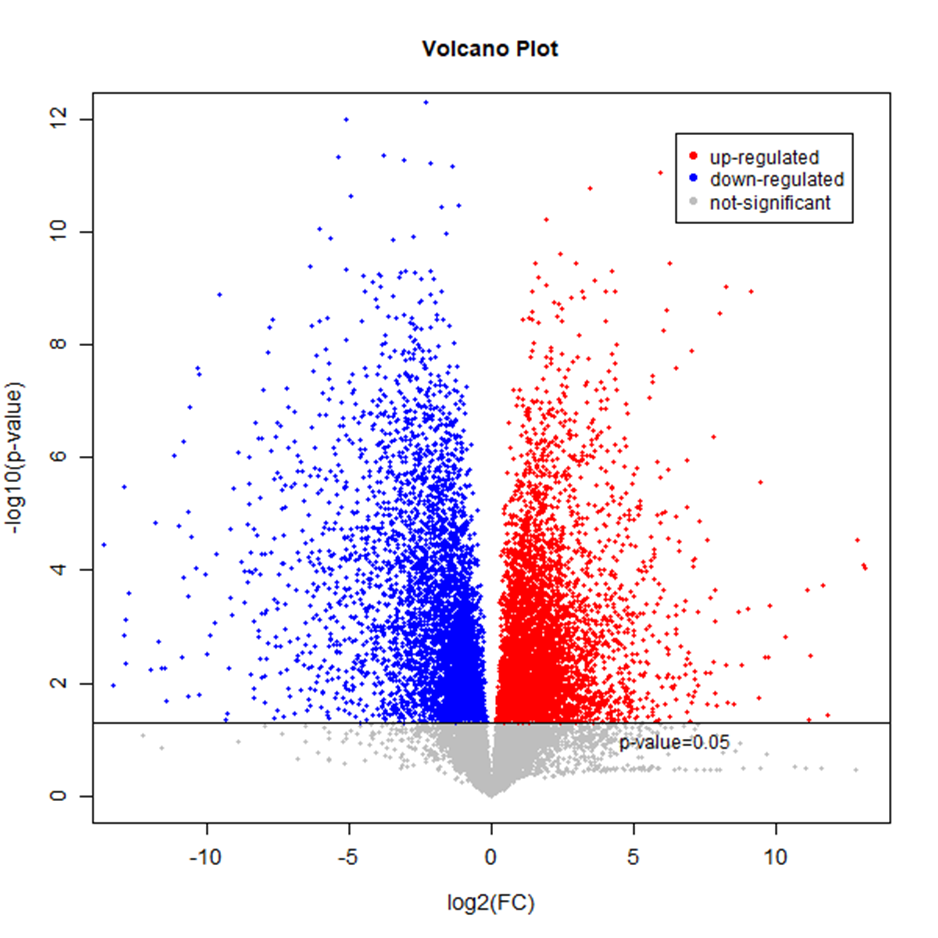


**E**
